# Genomic Diversity and Population Structure of Tibetan Antelopes Sampled From the Selincuo Region

**DOI:** 10.1002/ece3.74598

**Published:** 2026-11-10

**Authors:** Siwei Gao, Yibo Zhang, Zhonghao Yang, Xin Song, Wentao Kong, Yanling Zhao, Zili Ren

**Affiliations:** ^1^ College of Animal Science Xizang Agricultural and Animal Husbandry University Linzhi People's Republic of China; ^2^ State Key Laboratory of Microbial Technology Shandong University Qingdao Shandong People's Republic of China

**Keywords:** effective population size, genetic diversity, genome‐wide SNPs, linkage disequilibrium, population genetic structure, runs of homozygosity (ROH), Tibetan antelope

## Abstract

Based on genome‐wide SNP data, this study assessed genomic diversity and population structure in Tibetan antelopes sampled from the Selincuo National Nature Reserve. The analyses included runs of homozygosity (ROH), genomic inbreeding coefficients, genetic diversity, linkage disequilibrium (LD), effective population size, genomic relatedness, principal component analysis (PCA), and sNMF. Genomic inbreeding varied among the sampled individuals, with substantial variation among individuals. Short and medium‐length ROHs predominated, whereas long ROHs contributed little to total ROH coverage. This pattern indicates that population‐wide homozygosity is mainly associated with older shared ancestry, genetic drift, and historical demographic change, with limited evidence of severe recent inbreeding. Several individuals had relatively high ROH‐based inbreeding coefficients, indicating heterogeneous individual‐level autozygosity. The samples retained considerable genome‐wide SNP variation, although allele frequencies were unevenly distributed. PCA showed dispersion among individuals, while sNMF identified multiple ancestry components with substantial overlap in individual genetic backgrounds. Kinship analysis detected no first‐ or second‐degree relatives among the sampled individuals. Linkage disequilibrium declined with increasing physical distance between markers, providing a basis for estimating recent effective population size and evaluating demographic history. Overall, the analyzed individuals showed variation in genomic autozygosity, with several exhibiting elevated homozygosity. The LD‐based estimate of recent effective population size provides a baseline for continued genomic monitoring of Tibetan antelopes sampled from the Selincuo region. Broader geographic and temporal sampling is needed to assess population boundaries and changes in genetic diversity. Future work should expand sampling across geographic regions and time periods and compare multiple populations using genome‐wide differentiation, IBD sharing, isolation‐by‐distance analysis, demographic modeling, and direct estimates of migration or gene flow. These analyses will clarify historical demographic changes, recent effective population size variation, and genetic connectivity among protected areas.

## Graphical abstract

This study systematically assessed the inbreeding levels, genetic diversity, effective population size, and linkage disequilibrium of 57 Tibetan antelopes from the Selincuo Nature Reserve using whole‐genome SNP data. The results showed that the population exhibited low overall inbreeding, a homogeneous genetic structure, a high recent effective population size, and rapid short‐range linkage disequilibrium decay, indicating that distant mating effectively diluted inbreeding accumulation. However, the predominance of low‐frequency alleles and individual differences in inbreeding suggest potential risks. Regular monitoring of genetic diversity, expansion of gene flow across protected areas, and integration of multi‐omics data for management optimization are recommended.

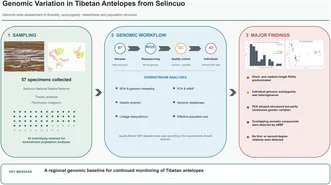

## 1 Introduction

The Tibetan antelope ( *Pantholops hodgsonii* ) is a rare wild ungulate endemic to the Qinghai–Tibet Plateau. It belongs to the genus Pantholops in the family Bovidae and is listed as a Class I protected animal in China. The species mainly inhabits cold deserts and alpine meadows at elevations of 3700–5500 m above sea level (Smith et al. 2010).

The Tibetan antelope exhibits seasonal migration. From May to July, females gather at wintering sites and migrate along established routes to traditional calving grounds. In August, they return with their young (Schaller 2000). These movements connect habitats across the plateau and highlight the importance of protecting migration routes and calving areas.

The Selincuo National Nature Reserve is located in the central part of the northern Tibetan Plateau and supports an important Tibetan antelope population. In recent years, the surface area of Selincuo Lake has expanded in association with climate change, potentially altering nearby grassland use and animal movements. Field surveys have also recorded repeated group activity by Tibetan antelopes in the reserve. These conditions make the population a suitable focus for genetic monitoring.

Historical illegal hunting associated with the trade in shahtoosh shawls caused a sharp decline in Tibetan antelope numbers and may have produced a severe population bottleneck. Stronger anti‐poaching measures and the establishment of national nature reserves subsequently supported population recovery. Genetic recovery can occur more slowly than demographic recovery after a bottleneck. The population may therefore retain elevated inbreeding, reduced genetic diversity, or a smaller effective population size. These features require molecular data for evaluation.

Previous genomic studies provide a foundation for population‐level analysis. Ge et al. (2013) produced a draft Tibetan antelope genome and identified gene‐family expansion and positive‐selection signals associated with high‐altitude adaptation. Xu et al. (2024) assembled the first high‐quality chromosome‐level reference genome for the species in 2024. This reference genome supports more detailed analyses based on whole‐genome SNP markers.

This study examined Tibetan antelopes sampled from the Selincuo National Nature Reserve using whole‐genome SNP data. The analyses evaluated genetic diversity, allele‐frequency composition, genomic autozygosity, inbreeding, linkage disequilibrium (LD), effective population size, genetic relatedness, and population structure in the analyzed sample set. The analytical framework included runs of homozygosity (ROH), heterozygosity, *F*_IS_, polymorphism information content, minor allele frequency, LD‐based effective population size estimation, genomic relationship analysis, principal component analysis (PCA), and sNMF‐based population structure analysis. ROH length and distribution provide information about the relative timing of autozygosity and shared ancestry (Ceballos et al. 2018; Purfield et al. 2012). LD patterns inform the interpretation of recombination, historical genetic drift, and effective population size, while marker properties and population structure can also affect these patterns (Waples and Do 2010; Waples and England 2011). Genomic relationship analysis provides a means of estimating relatedness among wild individuals when pedigree records are unavailable (VanRaden 2008).

This study characterized genomic variation, autozygosity, relatedness, and population structure among Tibetan antelopes sampled from the Selincuo region. The results provide a regional baseline for future genetic monitoring and for studies that delimit breeding populations and assess connectivity.

## 2 Materials and Methods

### 2.1 Sample Collection

Skin tissue samples from 57 naturally deceased *Pantholops hodgsonii* (Tibetan antelopes), which had died between 2020 and 2026, were preserved by wildlife conservation staff and collected on January 5, 2026, from Siling Co (Selincuo National Nature Reserve), Shenzha County, Nagqu City, Xizang Autonomous Region, China (30°28′48″ N, 89°4′54″ E). For each sample, a small piece of dried skin tissue was excised using fine scissors, the epidermal hair was removed, and the tissue was placed in a 10 mL centrifuge tube containing 75% ethanol for subsequent whole‐genome resequencing. But The breeding origins and migratory histories of individual animals were not recorded. Accordingly, geographic occurrence within the sampling area was used to define the regional sample set, without assigning the individuals to a single breeding population.

### 2.2 Ethics Statement

Before library construction, genomic DNA extracted from Tibetan antelope ( *Pantholops hodgsonii* ) samples was subjected to quality control. DNA integrity and potential RNA contamination were assessed by agarose gel electrophoresis. DNA purity was evaluated using a NanoDrop spectrophotometer based on the OD260/280 ratio, and DNA concentration was accurately measured with a Qubit fluorometer. Only samples with an OD260/280 ratio of 1.8–2.0, a total DNA amount greater than 1 μg, and no obvious degradation or RNA contamination were used for library construction. These criteria ensured that high‐quality DNA samples were used for sequencing, thereby reducing potential bias in library preparation, read mapping, and variant detection.

### 2.3 Data Quality Control and Library Construction

Qualified DNA samples were randomly fragmented to approximately 350 bp using a Covaris ultrasonicator. Paired‐end sequencing libraries were then prepared following the manufacturer's protocol, including end repair, A‐tailing, adapter ligation, purification, and PCR amplification. The constructed libraries were first quantified using Qubit 2.0 and diluted to 1 ng/μL. Insert size distribution was assessed with an Agilent 2100 Bioanalyzer, and the effective library concentration was determined by q‐PCR. Libraries with an effective concentration greater than 2 nM and the expected insert size distribution were selected for sequencing.

Sequencing was performed on the DNBSEQ‐T7 platform (MGI, Shenzhen, China) using a paired‐end strategy with a read length of 150 bp. The reference genome used for read alignment and SNP detection was the Tibetan antelope genome assembly NCBI_NWIPB_Pahodg_1.0, downloaded from the NCBI Genome database: https://www.ncbi.nlm.nih.gov/datasets/genome/?taxon=59538. Raw reads were filtered to remove adapter‐contaminated reads, reads with more than 5% unknown bases, and low‐quality reads, defined as reads in which bases with a quality score below 15 accounted for more than 40% of the read length. This filtering step was performed to minimize the influence of low‐quality sequences on downstream analyses, including read alignment and variant calling (Andersson et al. 2010; Chen et al. 2018). After filtering, raw reads, clean reads, valid data rate, Q20, Q30, GC content, and sequencing depth were calculated. For the 57 samples, the valid data rate ranged from 99.74% to 99.90%, Q20 from 98.60% to 99.22%, Q30 from 97.42% to 98.50%, GC content from 43.29% to 47.93%, and clean data volume from 28.25 to 45.18 Gb. After alignment to the reference genome, the average sequencing depth ranged from 8.65× to 14.08×, with 74.63%–92.17% of the genome covered by at least 5× depth. These results indicate that the sequencing data were reliable and provided sufficient coverage for subsequent SNP calling and population genetic analyses. Detailed quality control and mapping statistics are provided in Tables 1 and 2.

**TABLE 1:** Summary of whole‐genome resequencing data quality for Selincuo Tibetan antelope samples.

Quality metric	Number of samples	Mean	Minimum	Maximum	Description
Total reads	57	224,349,833	188,606,182	301,614,496	Number of raw sequencing reads per sample
Valid reads	57	100.00%	100.00%	100.00%	Proportion of reads retained after quality control
Duplicate reads	57	3.70%	1.79%	6.98%	Proportion of duplicated reads
Mapping rate	57	99.55%	95.82%	99.95%	Proportion of reads mapped to the reference genome
Mean coverage	57	10.39×	8.65×	14.08×	Average sequencing depth per sample
Genome covered at ≥ 5×	57	84.34%	74.63%	92.17%	Proportion of the genome covered by at least 5× depth
Genome covered at ≥ 10×	57	48.20%	33.51%	76.60%	Proportion of the genome covered by at least 10× depth
Genome covered at ≥ 20×	57	5.72%	2.08%	15.29%	Proportion of the genome covered by at least 20× depth

**TABLE 2:** Summary of raw sequencing data quality for Selincuo Tibetan antelope samples.

Quality metric	Number of samples	Mean	Minimum	Maximum	Description
Raw reads	57	224,349,833	188,606,182	301,614,496	Number of raw sequencing reads per sample
Raw bases	57	33.65 G	28.29 G	45.24 G	Total amount of raw sequencing bases per sample
Valid reads	57	224,349,833	188,606,180	301,614,496	Number of reads retained after quality control
Valid bases	57	33.60 G	28.25 G	45.18 G	Total amount of clean bases retained after quality control
Valid reads rate	57	99.83%	99.74%	99.90%	Proportion of reads retained after quality control
Q20 rate	57	98.95%	98.60%	99.22%	Proportion of bases with a quality score of 20 or higher
Q30 rate	57	98.05%	97.42%	98.50%	Proportion of bases with a quality score of 30 or higher
GC content	57	45.75%	43.29%	47.93%	Proportion of G and C bases in the sequencing data

### 2.4 Data Analysis

#### 2.4.1 Genomic Inbreeding Coefficient Analysis

To assess the sensitivity of genomic inbreeding estimates to the minimum ROH length threshold, ROH analyses were repeated using minimum lengths of 100, 300, 500, and 1000 kb. The 500‐kb threshold was used for the primary analysis, whereas the other thresholds were evaluated in a sensitivity analysis. These thresholds were selected to cover short, intermediate, and relatively long ROH segments. Previous studies have shown that the estimated proportion of the genome contained in ROH is strongly influenced by the minimum ROH length, marker density, and the algorithm used for ROH detection; therefore, results obtained under different thresholds should be compared rather than interpreted using a universal cutoff (McQuillan et al. 2008; Rosenberg et al. 2013).

All other ROH detection parameters were kept constant among analyses so that differences in the estimated genomic inbreeding coefficient could be attributed primarily to the minimum‐length criterion. ROHs were identified using PLINK v1.9 with a sliding window of 50 SNPs, allowing a maximum of one heterozygous genotype and five missing genotypes per window. Each ROH was required to contain at least 50 SNPs and to have a minimum marker density of one SNP per 50 kb. The ‐‐cow chromosome‐coding option was retained to ensure consistency with the primary analysis.

The analysis was performed using the autosomal dataset comprising 43 individuals and 6,991,680 SNPs distributed across chromosomes 1–29. The dataset was derived from the quality‐controlled genotype data and excluded sex chromosomes, mitochondrial sequences, unplaced scaffolds, and other non‐autosomal sequences. The total autosomal genome length used as the denominator was 2,566,700,306 bp, corresponding to the combined length of the 29 autosomes in reference genome GCA_040182635.1 (NWIPB_Pahodg_1.0).

(1) $$F_{\mathrm{ROH},j}=\frac{\sum_{k}L_{\mathrm{jk}}}{L_{\text{auto}}},$$

here *L*_*jk*_ is the physical length of the *k*th ROH and *L*_autol_ is the total length of autosomes 1–29 in the reference assembly. The autosomal genome length was obtained from the corresponding FASTA index file after validating the accession‐to‐chromosome mapping and was 2,566,700,306 bp. Individuals without ROH meeting the predefined thresholds were assigned *F*_ROH_ = 0.

ROH segments were grouped into six length classes: 0.5–1, 1–2, 2–4, 4–8, 8–16, and > 16 Mb. These classes were used to describe the relative contribution of ROH of different lengths to genomic inbreeding. In general, longer ROH segments are more consistent with relatively recent autozygosity, whereas shorter segments may reflect more distant common ancestry. However, ROH length classes were not converted into exact numbers of generations because such conversion requires a species‐specific recombination map and is also affected by demographic history, marker density, genotype error, and the ROH‐calling algorithm (Keller et al. 2011; Onzima et al. 2018).

For comparison, *F*_PLINK_ was estimated from the same autosomal dataset using the PLINK ‐‐het statistic:

(2) $$\begin{array}{c} F_{\text{PLINK}}=\left(O_{\mathrm{HOM}}-E_{\mathrm{HOM}}\right)/\left(N-E_{\mathrm{HOM}}\right) \end{array}$$

where *O*_HOM_ is the observed number of homozygous genotypes, *E*_HOM_ is the expected number of homozygous genotypes under Hardy–Weinberg expectations, and *N* is the number of non‐missing genotypes. The two estimates were merged by individual ID, and Pearson's correlation was calculated after confirming complete ID matching and the absence of missing values for all 43 individuals.

#### 2.4.2 ROH Sensitivity Analysis

To assess the sensitivity of genomic inbreeding estimates to the minimum ROH length threshold, we repeated the ROH analysis using minimum ROH lengths of 100, 300, 500, and 1000 kb. The 500‐kb threshold corresponded to that used in the primary analysis. All other ROH detection parameters were held constant across analyses to ensure that observed differences were attributable only to the minimum length criterion. ROHs were identified with PLINK v1.9 using a sliding window of 50 SNPs, allowing a maximum of one heterozygous site and five missing genotypes per window. Each ROH was required to contain at least 50 SNPs and to meet a minimum marker density of one SNP per 50 kb. The ‐‐cow chromosome coding option was retained to match the primary analysis.

Analyses were conducted using the autosomal dataset comprising 43 individuals and 6,991,680 SNPs located on chromosomes 1–29. The input dataset was derived from the quality‐controlled genotype dataset and contained no sex chromosomes, mitochondrial sequences, or unplaced scaffolds. The autosomal genome length used as the denominator was 2,566,700,306 bp, corresponding to the combined length of the 29 autosomes in the reference genome GCA_040182635.1 (NWIPB_Pahodg_1.0).

For individual *j*, the ROH‐based inbreeding coefficient was calculated as:

(3) $$\begin{array}{c} F_{\mathrm{ROH},j}=\frac{\sum_{k}L_{\mathrm{jk}}}{L_{\text{auto}}} \end{array}$$

where ∑*L*_ROH_ is the summed physical length of all ROH segments detected in that individual, and *L*_autosomes_ is the total autosomal genome length (2,566,700,306 bp). Individuals without detected ROHs would have been assigned *F*_ROH_ = 0; ROH were classified into six length categories: 0.5–1, 1–2, 2–4, 4–8, 8–16, and > 16 Mb. These categories were used as broad indicators of the relative contribution of older versus more recent autozygosity, because recombination generally fragments ancestral haplotypes over time and longer ROH therefore tend to reflect more recent common ancestry (Keller et al. 2011; Purcell et al. 2007). The categories were not interpreted as exact generation estimates, because ROH dating requires a species‐specific recombination map and is also affected by demographic history, marker density, and ascertainment.

For comparison, *F*_PLINK_ was calculated from the same autosomal dataset using PLINK ‐‐het as:

(4) $$\begin{array}{c} F_{\text{PLINK}}=\left(O_{\mathrm{HOM}}-E_{\mathrm{HOM}}\right)/\left(N-E_{\mathrm{HOM}}\right) \end{array}$$

where *F*_PLINK_ is the individual‐level inbreeding coefficient estimated by PLINK; *O*_HOM_ is the observed number of homozygous genotypes in the individual; *E*_HOM_ is the expected number of homozygous genotypes calculated from the allele frequencies in the dataset; and *N* is the total number of valid, non‐missing SNP genotypes used in the calculation. Thus, *N* − *E*_HOM_ represents the expected number of heterozygous genotypes. Positive values of *F*_PLINK_ indicate an excess of homozygous genotypes relative to expectation, whereas negative values indicate a relative excess of heterozygous genotypes.

The two estimates were merged by IID, and Pearsons correlation was calculated after confirming complete matching and non‐missing values for all 43 individuals. However, all 43 individuals had at least one ROH under each threshold. For each threshold, we calculated the total number of ROH segments, number of ROH segments per individual, total ROH length, mean and maximum segment lengths, and the mean and standard deviation of individual *F*_ROH_. Spearman rank correlations were calculated between individual *F*_ROH_ estimates obtained under each pair of minimum‐length thresholds to evaluate the consistency of relative inbreeding rankings.

#### 2.4.3 Population Genetic Diversity Analysis

Genetic diversity was quantified using the final autosomal SNP dataset comprising 6,991,680 high‐quality biallelic SNPs from 43 Tibetan antelope individuals. Genotype data were processed using PLINK v1.9.0, a widely used software package for genome‐wide genotype data management, quality control, and population genetic analyses (Purcell et al. 2007). The analysis was restricted to the retained individuals.

Variant‐ and sample‐level quality control was performed using a SNP missingness threshold of 5% (‐‐geno 0.05), an individual missingness threshold of 10% (‐‐mind 0.10), and a minor allele frequency threshold of 0.05 (‐‐maf 0.05). These thresholds were selected to reduce the potential effects of missing genotypes and low‐frequency variants while retaining a sufficiently large number of informative polymorphic markers. Similar missingness and minor allele frequency filters are commonly applied in genome‐wide SNP‐based population genetic and association analyses (Anderson et al. 2010). Variants located on chromosome 30, corresponding to the X chromosome under the bovine chromosome‐coding model used in PLINK, as well as variants located on unplaced scaffolds, were removed. Sites with missing, non‐standard, or identical allele annotations were subsequently excluded using a Python‐based filtering procedure. Only autosomal biallelic SNPs located on chromosomes 1–29 were retained for the genetic diversity analysis.

For each SNP, allele frequencies were estimated from non‐missing diploid genotypes. Let *n*_valid_ denote the number of individuals with non‐missing genotypes at a locus, and let *n*_homref_ and *n*_het_ denote the numbers of homozygous‐reference and heterozygous individuals, respectively. The reference‐allele frequency was calculated as

(5) $$\begin{array}{c} p=\left(2n_{\text{homref}}+n_{\mathrm{het}}\right)/\left(2n_{\text{valid}}\right), \end{array}$$

where *p* is the estimated frequency of the reference allele; *n*_homref_ is the number of individuals homozygous for the reference allele; *n*_het_ is the number of individuals heterozygous for the reference and alternative alleles; and *n*_valid_ is the total number of individuals with valid, non‐missing genotypes at the locus. The numerator, 2*n*_homref_ + *n*_het_, represents the total number of reference‐allele copies, whereas the denominator, 2*n*_valid_, represents the total number of allele copies in the sampled individuals.

The alternative‐allele frequency was defined as

(6) $$\begin{array}{c} q=1-p \end{array}$$

where *q* is the frequency of the alternative allele and *p* is the frequency of the reference allele. Because the two allele frequencies at a biallelic locus sum to 1, *q* is calculated as 1 − *p*. Thus, if *p* is the proportion of reference‐allele copies in the sampled individuals, *q* represents the remaining proportion of alternative‐allele copies.

Minor allele frequency was calculated as follows:

(7) $$\begin{array}{c} \mathrm{MAF}=\min\left(p,q\right) \end{array}.$$

where MAF is the minor allele frequency, and *p* and *q* are the frequencies of the two alleles at a biallelic locus. The function min(*p*,*q*) indicates that the smaller of the two allele frequencies is selected as the minor allele frequency. Thus, MAF ranges from 0 to 0.5.

Observed heterozygosity was calculated as the proportion of heterozygous individuals among individuals with non‐missing genotypes:

(8) $$\begin{array}{c} H_{O}=\frac{n_{\mathrm{het}}}{n_{\text{valid}}} \end{array}$$

where *H*_O_ is the observed heterozygosity; *n*_het_ is the number of individuals with heterozygous genotypes at the locus; and *n*_valid_ is the total number of individuals with valid, non‐missing genotypes at that locus. Thus, *H*_O_ represents the proportion of genotyped individuals that are heterozygous.

Expected heterozygosity was estimated under the Hardy–Weinberg equilibrium model:

(9) $$\begin{array}{c} H_{E}=2\mathrm{pq} \end{array}$$

In this study, *H*_O_ and *H*_E_ were calculated at the locus level. For each SNP, observed heterozygosity (Ho_locus) was calculated as the proportion of individuals carrying a heterozygous genotype among individuals with valid genotypes at that SNP. Expected heterozygosity (He_locus) was calculated as 2*pq*, where *p* and *q* are the allele frequencies at the same SNP. Thus, each *H*_O_ and *H*_E_ value represents one SNP locus.

For genome‐wide summaries, the mean and standard deviation of *H*_O_ and *H*_E_ were calculated across the per‐locus values obtained from all 6,991,680 analyzed autosomal SNPs. Therefore, the reported genome‐wide values represent summaries of the distribution of SNP‐level heterozygosity rather than averages of individual‐level heterozygosity. This approach is consistent with the use of locus‐based heterozygosity statistics to summarize genetic diversity across multilocus marker datasets (Nei 1973).

Polymorphism information content was calculated for each biallelic SNP according to Botstein et al. (1980):

(10) $$\begin{array}{c} \mathrm{PIC}=2\mathrm{pq}\left(1-\mathrm{pq}\right) \end{array}$$

where PIC is the polymorphism information content of the genetic marker; *p* and *q* are the frequencies of the two alleles at a biallelic locus, with their sum equal to 1 (*p* + *q* = 1); and (1*pq*) represents the probability that two randomly selected alleles are not both the same allele. A higher PIC value indicates greater genetic polymorphism and higher discriminatory power of the marker. For a biallelic marker, PIC ranges from 0 to 0.5, with the maximum value occurring when *p* = *q* = 0.5.

PIC was interpreted as a measure of marker informativeness based on allele‐frequency distribution and was reported as a complementary metric rather than as an independent substitute for *H*_O_ or *H*_E_. For each metric, the arithmetic mean, standard deviation, median, quartiles, minimum, and the number of loci with finite values were calculated across the retained SNPs. All calculations used the complete autosomal SNP panel; no visualization‐based subsampling was used to generate the numerical summaries.

#### 2.4.4 Principal Component Analysis

PCA was performed using PLINK v1.9.0‐b.7.11 to assess genome‐wide genetic structure among the Tibetan antelope individuals. PLINK is widely used for genome‐wide genotype quality control, population structure analysis, and PCA (Purcell et al. 2007). The analysis was conducted using the ‐‐pca 20 option, which extracts the first 20 principal components from the genotype data.

The input dataset comprised 733,013 LD‐pruned autosomal SNPs. Before LD pruning, variants were filtered using a minor allele frequency threshold of 0.05, a genotype missingness threshold of 5%, and an individual missingness threshold of 10%. These quality‐control criteria were applied to reduce the influence of rare variants, incomplete genotypes, and samples or markers with high missingness on estimates of genetic structure (Anderson et al. 2010). LD pruning was then performed using the PLINK command ‐‐indep‐pairwise 50 10 0.2, with a window size of 50 variants, a step size of 10 variants, and an *r* ^2^ threshold of 0.2. LD pruning reduces the overweighting of highly correlated markers and helps ensure that PCA is not disproportionately influenced by clusters of linked SNPs (Price et al. 2006).

Hardy–Weinberg equilibrium filtering was not applied before PCA. This decision was made because deviations from Hardy–Weinberg equilibrium may reflect genuine population structure, admixture, demographic history, inbreeding, or other biological processes. Removing such variants solely on the basis of HWE deviations could therefore eliminate informative loci or bias the assessment of population structure, particularly when the sampled individuals may originate from more than one genetic group (Wigginton et al. 2005).

Non‐autosomal variants, including variants located on chromosome 30, which corresponds to the X chromosome under the bovine chromosome‐coding system used in PLINK, as well as variants located on unplaced scaffolds, were excluded before LD pruning. Consequently, all 733,013 SNPs retained after LD pruning were autosomal and were used for PCA. PCA was conducted on the filtered dataset comprising 43 individuals after the removal of putative duplicate or highly related samples.

PLINK calculated principal components from the genotype‐derived covariance or relationship structure among individuals. The eigenvectors generated by PLINK were used as individual PC scores, whereas the corresponding eigenvalues were used to quantify the amount of genetic variation represented by successive components (Patterson et al. 2006). The first 20 principal components were retained for downstream interpretation. Scatterplots of PC1 against PC2 were generated to visualize broad patterns of genetic similarity and differentiation among individuals.

#### 2.4.5 Population Genetic Structure Analysis Using sNMF

The genetic structure of 43 Tibetan antelope individuals was estimated using the sNMF method implemented in the LEA package. The input data were derived from 733,013 LD‐pruned SNPs in the unified autosomal dataset. To reduce computational demands, the input VCF file was thinned to approximately 97,159 SNPs for the sNMF analysis. All markers were located on autosomes 1–29, and the sample order was kept consistent with that used in the PCA.

The sNMF analysis began with conversion of the VCF file into the .geno format required by LEA. The VCF file contained genotype data for 43 individuals. Conversion was performed using R 4.5.2 and LEA v3.22.0, with the random seed fixed at 12,345.

Biallelic SNPs located on autosomes 1–29 were retained during conversion. Variants with multiple ALT alleles, REF or ALT alleles outside A, C, G, and T, or REF and ALT sequences longer than 1 bp were excluded. For each marker, missing genotypes were omitted when genotype variation was assessed. Monomorphic markers with only one observed non‐missing genotype were removed. The retained markers were transposed and written to the input.geno file, which was formatted with one SNP per row and one individual per column. Genotypes were encoded as 0, 1, 2, or 9.

The sNMF models were run using LEA v3.22.0. The number of ancestral components, *K*, ranged from 2 to 8, with 10 independent repetitions performed for each *K* value. A total of 70 model runs were completed using four CPU cores and a fixed random seed of 12,345.

The models estimated individual genetic‐component coefficients through sparse non‐negative matrix factorization. Each row of the *Q* matrix summed to 1, and each *Q* value represented the estimated proportion of an individual assigned to the corresponding component. The default sparsity parameter in LEA was used. The convergence threshold and maximum number of iterations followed the software defaults.

Model performance was evaluated using cross‐entropy for each value of *K*. During cross‐entropy estimation, a proportion of the genotype data was randomly masked, and the remaining genotypes were used to fit the model. The fitted model then predicted the masked genotypes. Lower cross‐entropy values indicated better predictive performance under the evaluated model. Cross‐validation and predictive‐error criteria are commonly used to compare alternative genetic‐structure models, although the specific implementation here followed the LEA software procedure.

For each *K* value, the best cross‐entropy, mean cross‐entropy, and standard deviation were calculated across the 10 repetitions, and the repetition corresponding to the lowest cross‐entropy was recorded. The mean cross‐entropy was used as the primary criterion for model selection, with the single best value and median value also reported.

The *Q* matrix from the repetition with the lowest cross‐entropy was extracted for each *K* value. The matrices were organized according to individual identity, with sample identifiers and dominant‐component assignments added. The dominant component was defined as the component with the highest *Q* value in each individual.

The *Q* matrices for *K* = 2 to *K* = 8 were saved as Q_matrix_K2.csv to Q_matrix_K8.csv. Cross‐entropy results were saved in ce_all_runs.csv, which included the *K* value, repetition number, cross‐entropy value, and run status.

The sNMF bar plots were generated in R to display the *Q*‐matrix composition of the 43 individuals for *K* = 2 to *K* = 8. Each individual was represented by one stacked bar, with different colors denoting different genetic components. The vertical axis represented *Q* values. Individuals were ordered first according to their dominant component and then according to the proportion assigned to that component.

Separate ancestry‐composition plots were also generated for *K* = 2 to *K* = 8, together with a plot showing the change in cross‐entropy across *K* values. All figures were exported at a resolution of 300 dpi.

The sNMF analysis was conducted in the remote server directory /home/GSW001029/snmf_final. The computational environment consisted of R 4.5.2 and LEA v3.22.0, with four CPU cores and a fixed random seed of 12,345. The input dataset contained 43 individuals and approximately 97,159 SNPs. The reference genome was *Pantholops hodgsonii* NCBI_NWIPB_Pahodg_1.0, accession number GCA_002942975.1.

#### 2.4.6 Kinship Analysis

Pairwise relatedness was assessed using genome‐wide SNP data and quantified from identity‐by‐descent estimates obtained with *P*_LINK_ (Purcell et al. 2007). The analysis used the 43 individuals retained after the preliminary relatedness screening. A total of 733,013 LD‐pruned autosomal SNPs from chromosomes 1–29 were used for the final kinship analysis. LD pruning was performed with PLINK v1.9.0‐b.7.11 using the ‐‐indep‐pairwise 50 10 0.2 option. This procedure used a sliding window of 50 SNPs, advanced the window by 10 SNPs at each step, and retained 733,013 SNPs for downstream analysis. The remaining 6,258,667 SNPs were excluded from the LD‐pruned dataset. The genotype call rate of the retained dataset was 99.6961%.

The LD‐pruned SNP set was extracted from the unified dataset using PLINK. The resulting binary files contained 733,013 SNPs and 43 individuals. Pairwise IBD estimates were then calculated with the PLINK ‐‐genome full option. The analysis included all 903 unique pairwise comparisons, calculated as:

(11) $$\begin{array}{c} N_{\text{pairs}}=\frac{n\left(n-1\right)}{2}=\frac{43\times 42}{2}=903. \end{array}$$

Pairwise relationships were classified according to PI_HAT values. Pairs with PI_HAT values of at least 0.80 were classified as putative duplicates or monozygotic twins. Values from 0.40 to 0.80 represented first‐degree relationships, values from 0.125 to 0.40 represented second‐degree relationships, and values below 0.125 were classified as unrelated.

#### 2.4.7 Linkage Disequilibrium Analysis

LD decay was analyzed using the same autosomal SNP dataset applied in the genetic diversity analyses. The original genotype dataset contained 12,747,261 SNPs from 57 individuals. Pairwise relatedness analysis identified 14 highly related individuals, which were removed before the primary analyses. The remaining 43 individuals were extracted using PLINK v1.9, and allele frequencies and missingness were recalculated within this subset. Variants with a minor allele frequency below 0.05, genotype missingness above 5%, or individual missingness above 10% were removed. This filtering produced a quality‐controlled dataset containing 7,128,553 SNPs (Purcell et al. 2007; Anderson et al. 2010).

The quality‐controlled dataset contained markers assigned to autosomes 1–29, chromosome 30, which corresponds to the X chromosome under the bovine chromosome coding system, and additional scaffold sequences. For the LD analysis, we retained only biallelic SNPs located on autosomes 1–29 with alleles represented by A, C, G, or T. This filtering was performed using PLINK v1.9.0‐b.7.11 64‐bit, released on August 19, 2025, with the ‐‐cow, ‐‐chr 1–29, and ‐‐snps‐only just‐acgt options. The resulting autosomal dataset contained 6,991,680 SNPs. In total, 129,772 SNPs assigned to chromosome 30, 4221 scaffold‐associated SNPs, and 2880 sites with nonstandard allele representations were removed. The retained dataset provided a consistent diploid autosomal marker panel for pairwise LD estimation and subsequent *N*_e_ calculations.

No additional LD pruning was applied before LD estimation. LD pruning would reduce the physical‐distance‐dependent correlations that are required for estimating LD decay. To reduce the computational burden associated with pairwise calculations, approximately 2% of the 6,991,680 autosomal SNPs were randomly selected using the PLINK ‐‐thin 0.02 option. Three independent thinning replicates were generated using fixed random seeds of 1001, 2002, and 3003. The resulting datasets contained 140,021, 139,969, and 140,336 SNPs, respectively. The same 43 individuals were retained in all replicates.

Pairwise LD was calculated using the unphased genotype dosage *r* ^2^ statistic implemented in *P*_LINK_. Only intrachromosomal SNP pairs separated by no more than 1000 kb were retained. The SNP‐count window was set to 99,999, and the minimum *r* ^2^ threshold was set to zero, allowing pairs with *r* ^2^ = 0 to be included. Cross‐chromosomal SNP pairs were not analyzed. For each replicate, the physical distance between paired SNPs was divided into 190 bins. Distances from 0 to 100 kb were divided into 1‐kb intervals, and distances from 100 to 1000 kb were divided into 10‐kb intervals. Finite *r* ^2^ values were summarized within each bin. A minimum of 10 valid SNP pairs per replicate was required, and a bin was retained when at least two of the three replicates met this criterion. The primary LD estimate for each bin was calculated as the equal‐weighted mean of the replicate‐specific means. Pair‐count‐weighted means were calculated as a sensitivity analysis.

#### 2.4.8 Estimation of LD‐Based Effective Population Size

Effective population size was estimated from the pooled mean *r* ^2^ value for each retained distance bin. The finite‐sample correction was calculated according to

(12) $$\begin{array}{c} r_{\mathrm{adj}}^{2}=r_{\mathrm{obs}}^{2}-\frac{1}{2n} \end{array},$$

where *n* represents the number of sampled diploid individuals. With 43 individuals, the correction was 1/(2*n*) = 1/86 = 0.011628. This correction was applied to the pooled mean *r* ^2^ value for each distance bin. Bins with nonfinite values or corrected *r* ^2^ values less than or equal to zero were excluded from the *N*_e_ calculation.

Following Hill (1981), effective population size was estimated using

(13) $$\begin{array}{c} N_{e}=\frac{1/r_{\mathrm{adj}}^{2}-1}{4c} \end{array}$$

where *r* ^2^_adj_ is the corrected *d* LD estimate and *c* is the recombination distance in Morgans. Physical distance was converted to recombination distance according to

(14) $$\begin{array}{c} c=d\times R\times 10^{-5} \end{array}$$

where *d* is the physical distance in kb and R is the assumed recombination rate in cM/Mb. The primary analysis used a recombination rate of 1.0 cM/Mb as a cattle‐based proxy because a species‐specific genetic map was unavailable. The corresponding recombination timescale was calculated as:

(15) $$\begin{array}{c} t=\frac{1}{2c} \end{array}$$

These values represent model‐dependent generations associated with the assumed recombination distance. They were not converted into calendar years because an appropriate generation interval for the sampled Tibetan antelope population was unavailable.

The effect of the recombination‐rate assumption was assessed using rates of 0.5, 0.8, 1.0, 1.2, and 1.5 cM/Mb. The same corrected *r* ^2^ values were used across these analyses, while the physical‐to‐genetic distance conversion was changed according to the tested recombination rate. The LD calculation, distance‐bin summarization, finite‐sample correction, and *N*_e_ estimation were performed using PLINK v1.9.0‐b.7.11 and custom Python scripts. The scripts were run with Python 3.13.12 using NumPy 2.4.3, pandas 3.0.1, and matplotlib 3.10.8.

The three thinning replicates were used to evaluate the reproducibility of the LD and *N*_e_ estimates under random marker subsampling. The reported replicate standard deviations describe variation among the three thinning datasets. They do not represent formal confidence intervals or bootstrap intervals. A display ceiling of 50,000 was applied to the plotted *N*_e_ values. Values exceeding this ceiling were retained in the output files and marked as above the display limit. LOWESS smoothing was performed using a custom implementation with tricube weighting, local linear regression, and frac = 0.3. The smoothed curves were used for visualization only, and all numerical *N*_e_ estimates were calculated from the unsmoothed bin‐level *r* ^2^ values.

## 3 Results

### 3.1 Runs of Homozygosity and Inbreeding Coefficient

#### 3.1.1 Runs of Homozygosity

In Table 3 the overall ROH‐based inbreeding level among the sampled Geladandong Tibetan antelopes appeared to be relatively low, with clear variation among individuals. When ROHs of at least 500 kb were included, *F*_ROH_ ranged from 0.0022 to 0.0351, with a mean of 0.0114, a median of 0.0081, and a standard deviation of 0.0082 (Figure 1). Most individuals had *F*_ROH_ values close to 0.003 to 0.010. A small number of individuals showed higher values, extending the distribution to 0.0351. The mean exceeded the median, suggesting that the higher inbreeding coefficients were concentrated in a limited number of individuals. Because the samples were obtained from naturally deceased animals, the observed *F*_ROH_ distribution may reflect the composition of the sampled individuals and should be interpreted within this sampling context.

**TABLE 3:** Distribution of ROH segments and their contributions to genomic inbreeding across ROH length classes.

Category	Number of ROH segments	Total length (Mb)	Contribution to mean *F*_ROH_
All detected ROH (≥ 0.5 Mb)	1499	1254.4	0.0114
0.5–1.0 Mb	1190	814.5	0.0074
1.0–2.0 Mb	273	355.4	0.0032
2.0–4.0 Mb	36	84.4	0.0008
4.0–8.0 Mb	0	0.0	0.0000
8.0–16.0 Mb	0	0.0	0.0000
> 16.0 Mb	0	0.0	0.00

**FIGURE 1:**
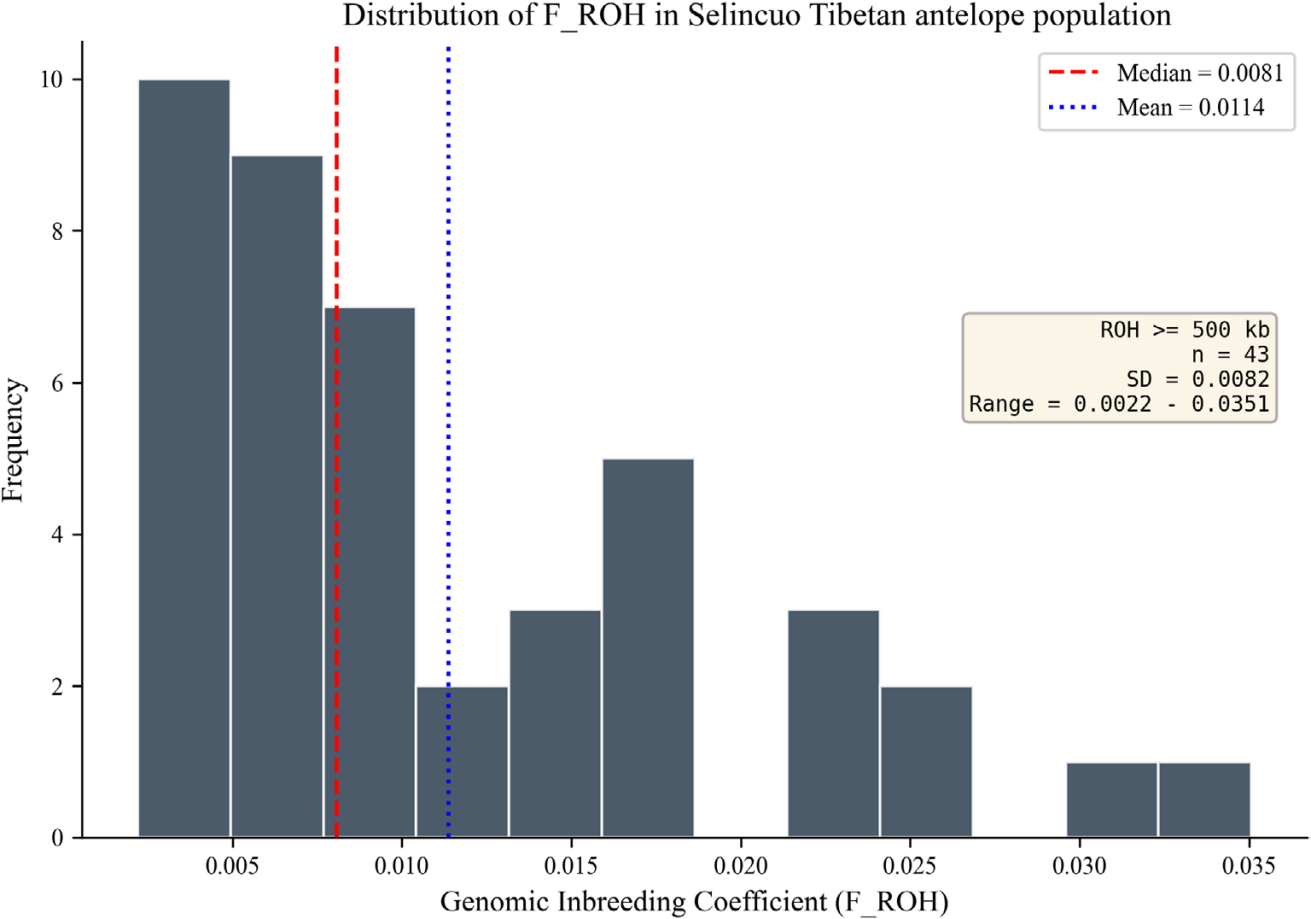
Distribution of genomic inbreeding coefficients estimated from runs of homozygosity (*F*_ROH_) in 43 Tibetan antelope individuals. The histogram shows the frequency distributionof individual *F*_ROH_ values calculated from ROH ≥ 500 kb. The red dashed line indicates the median *F*_ROH_ value (0.0081), and the blue dotted line indicates the mean value (0.0114).

#### 3.1.2 Sensitivity Analysis of *F*_ROH_

In Table 4 the minimum ROH length threshold had a clear effect on the estimation of *F*_ROH_. When the threshold was set at 1000, 500, 300, and 100 kb, the individual *F*_ROH_ values ranged from 0.0004 to 0.0156, 0.0022 to 0.0351, 0.0063 to 0.0466, and 0.0346 to 0.0827, respectively. ROH counts and cumulative ROH coverage increased as the threshold decreased, indicating that short ROHs accounted for a considerable proportion of the homozygous regions in individual genomes. ZLY_19, ZLY_28, ZLY_40, ZLY_42, and ZLY_5 showed relatively high *F*_ROH_ values under several thresholds. ZLY_28 showed a marked increase, from 0.0156 at the 1000 kb threshold to 0.0827 at the 100 kb threshold. Its consistently high values across thresholds suggest that both long and short ROHs contributed to its elevated genomic homozygosity. ROHs longer than 1000 kb are generally associated with common ancestors from more recent generations, whereas shorter ROHs are more likely to reflect older shared ancestry and historical genetic background. Across the different thresholds, genomic homozygosity among the sampled individuals was influenced mainly by short‐ to medium‐length ROHs, whereas very long ROHs made a limited contribution. While the overall contribution of very long ROHs remained relatively limited.

**TABLE 4:** Sensitivity of runs of homozygosity (ROH)‐based genomic inbreeding estimates to the minimum ROH length threshold in 43 Tibetan antelope individuals.

Minimum ROH length	ROH segments	Mean segments per individual	Mean *F*_ROH_	Median *F*_ROH_	SD	Range
100 kb	28,806	669.9	0.0530	0.0519	0.0121	0.0346–0.0827
300 kb	3669	85.3	0.0188	0.0154	0.0101	0.0063–0.0466
500 kb	1499	34.9	0.0114	0.0081	0.0082	0.0022–0.0351
1000 kb	309	7.2	0.0040	0.0019	0.0043	

#### 3.1.3 Individual Inbreeding Levels Estimated by *F*_PLINK_ and Their Variation

The PLINK‐based inbreeding coefficients showed substantial variation among individuals (Figure 2). *F*_PLINK_ ranged from −0.0142 to 0.1447, with a mean of 0.0625, a median of 0.0605, and a standard deviation of 0.0360. Most estimates fell between 0.03 and 0.10. A small number of individuals had values above 0.10, while several individuals had values close to zero or below zero. This distribution indicates variation in relative genomic homozygosity among the sampled individuals. Some individuals had observed homozygosity above the level expected from the allele frequencies in the sample. Estimates of *F*_PLINK_ can also be affected by allele‐frequency distributions, population structure, and marker composition, so the statistic is mainly useful for describing differences in homozygosity relative to the sampled population. In the present dataset, *F*_PLINK_ therefore describes relative differences in homozygosity among the sampled individuals.

**FIGURE 2:**
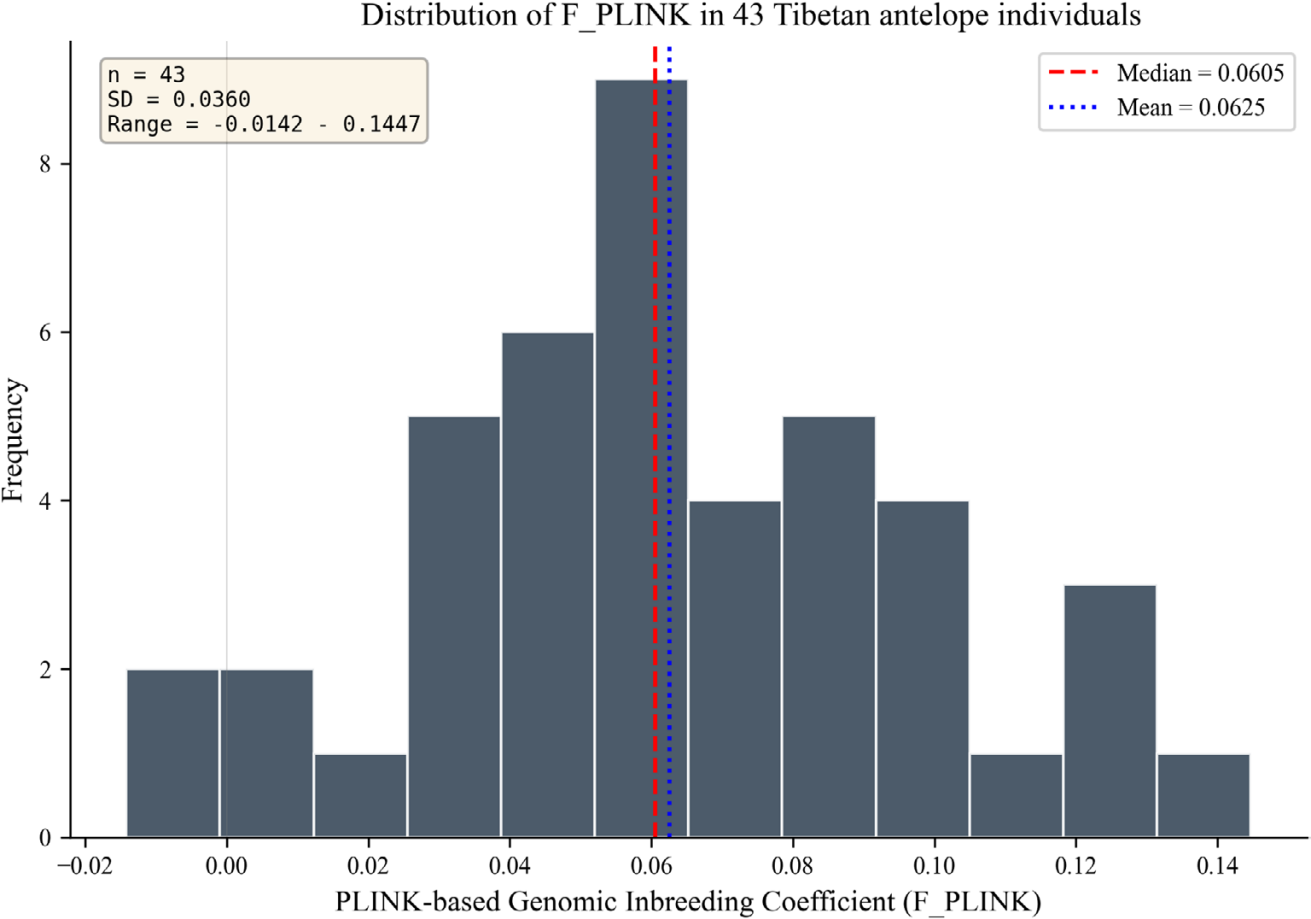
Distribution of PLINK‐based genomic inbreeding coefficients (*F*_PLINK_) in 43 Tibetan antelope individuals. The histogram shows the frequency distribution of individual estimates, including both negative and positive values. The dashed red line indicates the median value (0.0606), and the dotted blue line indicates the mean value (0.0625). Negative estimates are retained as calculated by PLINK and are not interpreted independently of the underlying allele‐frequency structure.

The *F*_ROH_ and *F*_PLINK_ results both indicate variation in genomic homozygosity among individuals in the Geladandong Tibetan antelope population. The mean *F*_ROH_ was 0.0114 under the 500 kb threshold, suggesting that long ROHs covered a relatively small proportion of the genome overall. The marked increase in *F*_ROH_ after shorter fragments were included indicates a substantial contribution from short ROHs to total homozygosity. The mean *F*_PLINK_ was 0.0625, which is consistent with relatively high homozygosity in some individuals. Taken together, the sample may have a relatively low level of recent inbreeding, while several individuals show higher genomic homozygosity. This variation appears to involve ROHs across multiple length classes.

### 3.2 Population Genetic Diversity Analysis

#### 3.2.1 Minor Allele Frequency (MAF) Distribution

The minor allele frequency (MAF) distribution was examined in the unpruned and LD‐pruned SNP datasets (Figure 3). The unpruned dataset contained 6,991,680 SNPs, and the LD‐pruned intersection subset contained 1,035,920 SNPs. Both datasets included many SNPs with relatively low MAF values, followed by a gradual decrease across the intermediate frequency range. The median per‐locus MAF was 0.1628 in the unpruned dataset and 0.1279 in the LD‐pruned dataset.

**FIGURE 3:**
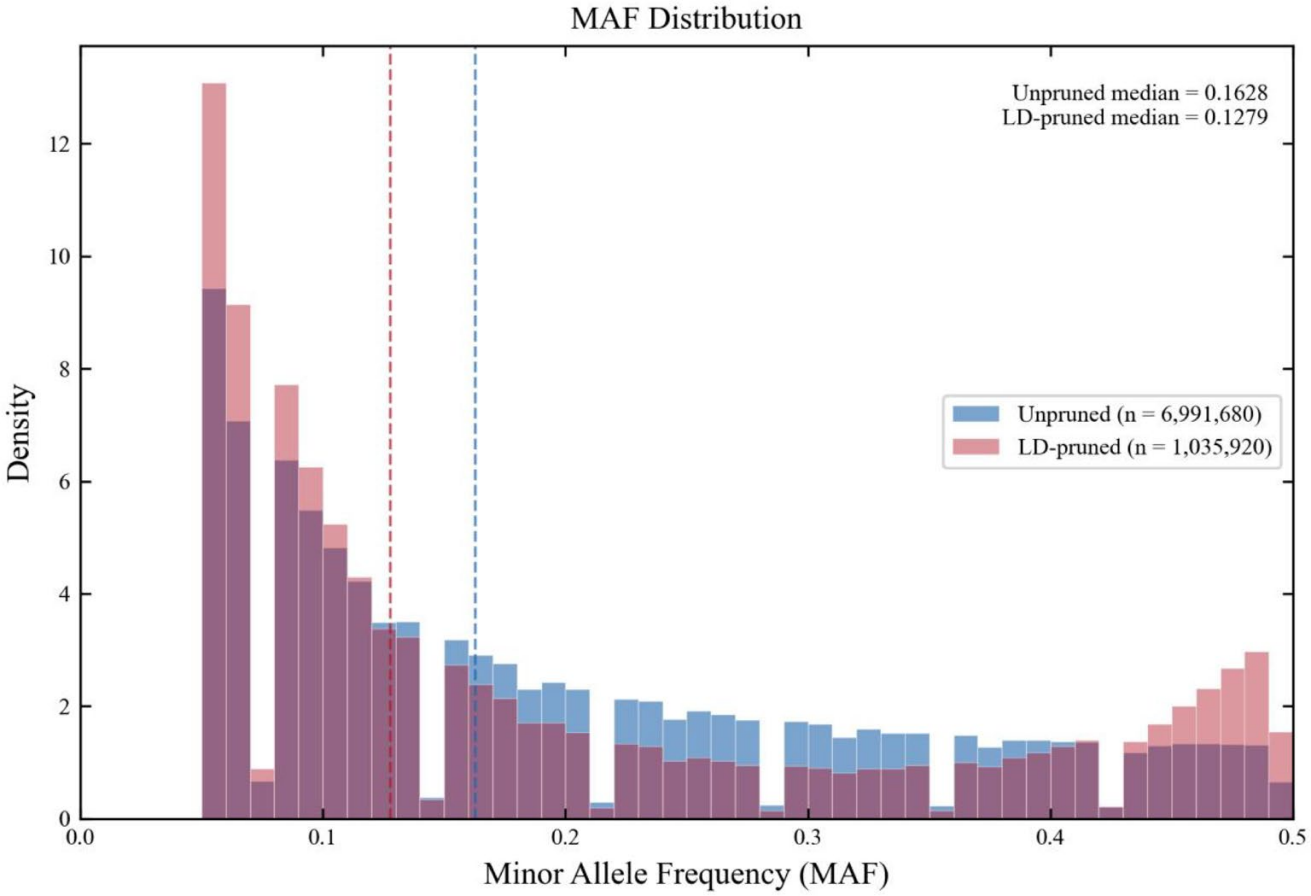
Distribution of minor allele frequencies (MAFs) in the unpruned and LD‐pruned SNP datasets. The blue and pink histograms represent the unpruned dataset containing 6,991,680 SNPs and the LD‐pruned dataset containing 1,035,920 SNPs, respectively. Dashed lines indicate the median MAF values of the unpruned (0.1628) and LD‐pruned (0.1279) datasets.

The two datasets showed broadly similar distributions. The LD‐pruned dataset contained a relatively larger proportion of low‐frequency variants, while the unpruned dataset retained more variants in the intermediate and higher frequency ranges. This pattern may reflect changes in SNP composition associated with LD pruning. Overall, the observed distributions covered a broad range of allele frequencies and provided a useful marker set for subsequent population genomic analyses.

#### 3.2.2 Heterozygosity Analysis

Observed and expected heterozygosity were summarized across the genome‐wide SNP dataset and compared between the unpruned and LD‐pruned datasets (Figure 4). All heterozygosity and *F*_IS_ values reported here are locus‐level estimates. One *H*_O_, *H*_E_, and *F*_IS_ value was calculated for each analyzed SNP, and the reported means and standard deviations summarize the distributions of these per‐locus estimates.

**FIGURE 4:**
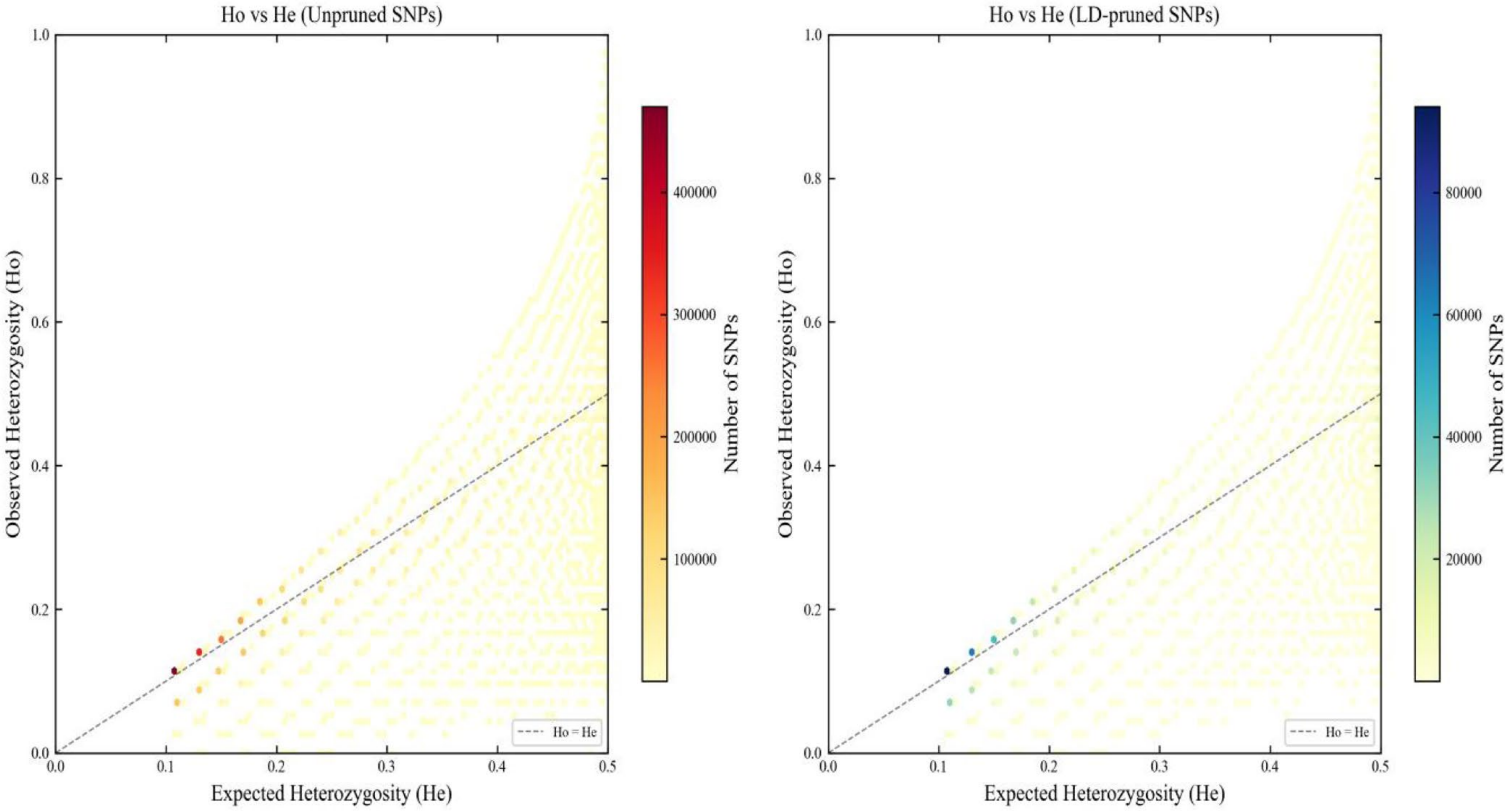
Relationship between oObserved and expected heterozygosit across SNPs in the unpruned and LD‐pruned datasets. The left and right panels show the results for the unpruned and LD‐pruned SNP datasets, respectively. Each point represents a binned combination of observed heterozygosity (*H*_O_) and expected heterozygosity (*H*_E_), and point color indicates the number of SNPs within each bin. The dashed gray line represents the expected relationship *H*_O_ = *H*_E_. The unpruned dataset contained 6,991,680 SNPs, whereas the LD‐pruned intersection subset contained 1,035,920 SNPs.

The primary unpruned dataset contained 6,991,680 autosomal SNPs. Across these loci, the mean observed heterozygosity was 0.2715 (SD = 0.1358), and the mean expected heterozygosity was 0.2897 (SD = 0.1330). The corresponding median values were 0.2558 and 0.2726. Observed heterozygosity was therefore lower than expected heterozygosity at both the mean and median levels. The distributions were broad, with observed heterozygosity ranging from 0 to 1.0000 and expected heterozygosity ranging from 0.1095 to 0.5000.

The joint distribution of *H*_O_ and *H*_E_ placed most loci below the *H*_O_ = *H*_E_ reference line, which was consistent with lower observed heterozygosity across many SNPs. A similar pattern appeared in the LD‐pruned dataset, where the median values were 0.2093 for *H*_O_ and 0.2231 for *H*_E_. The mean per‐locus *F*_IS_ was 0.0591, and the median was 0.0315. These values suggest a modest heterozygosity deficit relative to Hardy–Weinberg expectations. The interpretation remains subject to the effects of population structure, relatedness among samples, allele‐frequency composition, sampling design, and genotyping characteristics.

#### 3.2.3 Polymorphism Information Content (PIC)

The PIC distribution was used to describe the informativeness of the SNP markers (Figure 5). Among the 6,991,680 unpruned SNPs, PIC values ranged from 0.1035 to 0.3750. The mean PIC was 0.2389 (SD = 0.0930), and the median was 0.2354. The interquartile range extended from 0.1545 to 0.3307, indicating variation in marker informativeness across loci.

**FIGURE 5:**
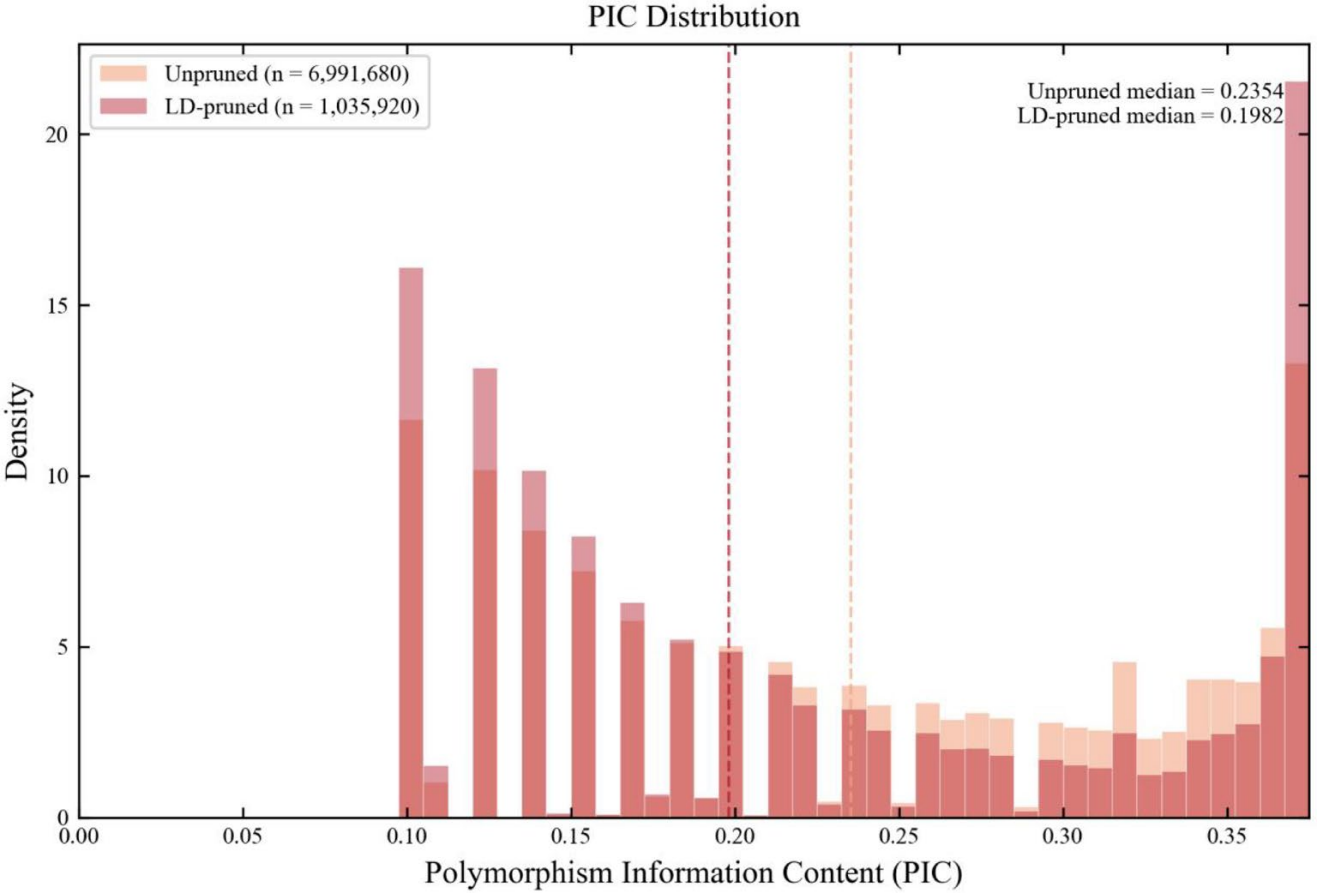
Distribution of polymorphism information content (PIC) values in the unpruned and LD‐pruned SNP datasets. The light‐orange and dark‐pink histograms represent the unpruned dataset containing 6,991,680 SNPs and the LD‐pruned dataset containing 1,035,920 SNPs, respectively. Dashed vertical lines indicate the median PIC values for the unpruned (0.2354) and LD‐pruned (0.1982) datasets.

The median PIC in the LD‐pruned dataset was 0.1982. This lower value may be related to the changed allele‐frequency composition of the pruned marker panel. The relatively low PIC values were consistent with the high proportion of low‐frequency variants observed in the SNP dataset. Such variants generally provide less information for individual‐marker analyses, which may account for the comparatively low marker informativeness. PIC was consequently used as a descriptive statistic for marker informativeness and was interpreted together with the heterozygosity measures when describing the SNP dataset. The PIC distribution was not taken as a direct measure of overall genetic diversity, and low PIC values were not interpreted as evidence of low genetic diversity.

#### 3.2.4 Principal Component Analysis

In Figure 6 PCA was performed using the relatedness‐filtered SNP dataset. PC1 and PC2 explained 7.3% and 5.6% of the total genetic variation, respectively. The individuals occupied several relatively compact regions of the PCA space, with a smaller number located near the margins of these regions.

**FIGURE 6:**
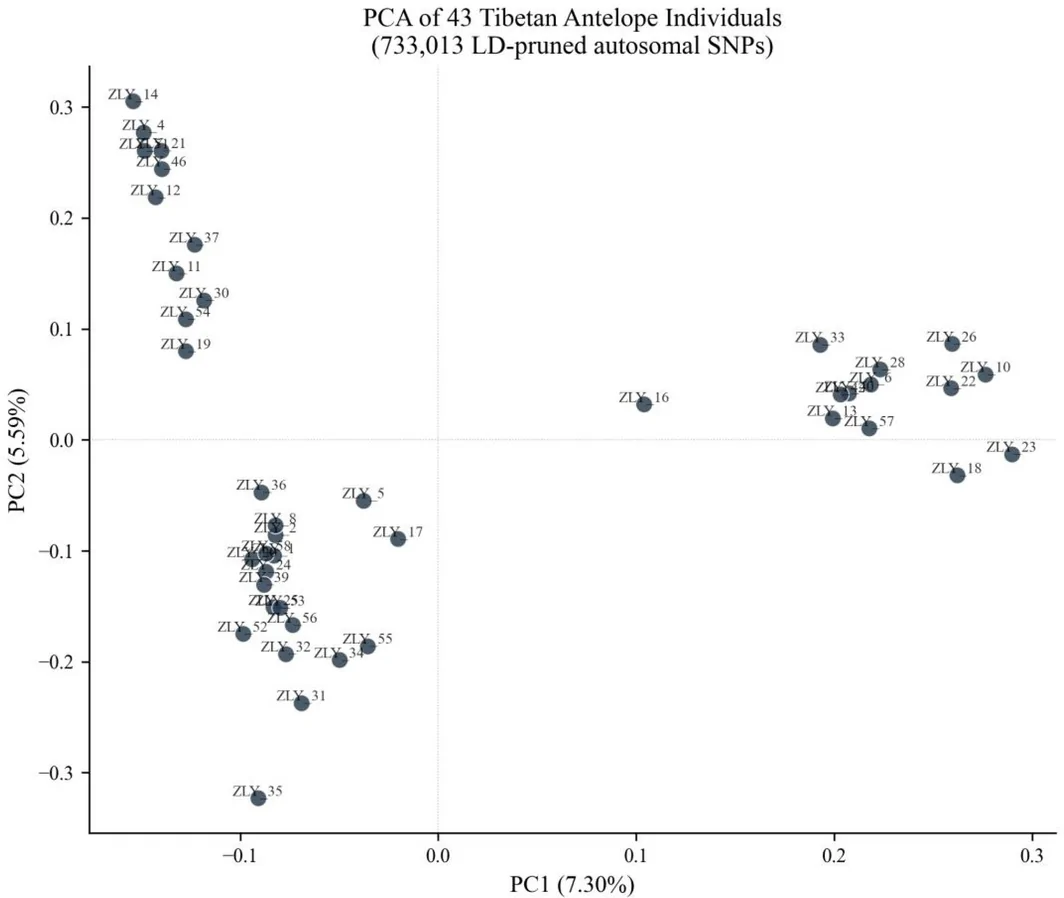
Principal component analysis (PCA) of Tibetan antelope individuals based on the relatedness‐filtered autosomal SNP dataset. PC1 and PC2 explained 7.3% and 5.6% of the total genetic variation, respectively. The samples were distributed across several localized regions of the PCA space, with most individuals forming relatively compact clusters near negative PC1 values and around PC1 values of 0.19–0.29. Overall, the PCA revealed a structured but partly continuous pattern of genetic variation, with differentiation occurring along both PC1 and PC2 rather than forming two completely discrete groups.

The main distribution extended along PC1 from negative to positive values. Four concentrations were apparent, with individuals located approximately at PC1 values of −0.14 to −0.10, −0.10 to −0.07, −0.05 to −0.02, and 0.19 to 0.29. One additional individual occurred near PC1 = 0.10 and PC2 = 0.03. Individuals with negative PC1 values covered both positive and negative PC2 values, while those with positive PC1 values were concentrated near PC2 = 0.00.

The PCA indicated a structured yet partly continuous pattern of genetic variation. Several localized concentrations were present, but the samples did not form two clearly separated groups. The main differentiation occurred along PC1. Variation along PC2 was more evident among individuals with negative PC1 values.

### 3.3 Population Genetic Structure Analysis Using sNMF

Genetic structure within the analyzed regional sample set was examined using the sparse non‐negative matrix factorization (sNMF) approach by testing values of *K* from 2 to 8 (Figure 7). The cross‐entropy value was lowest at *K* = 2 (0.5000) and increased with higher values of *K*, reaching 0.5305, 0.5538, 0.5846, 0.6074, 0.6334, and 0.6517 at *K* = 3, *K* = 4, *K* = 5, *K* = 6, *K* = 7, and *K* = 8, respectively. Among the tested models, *K* = 2 showed the lowest cross‐entropy value and summarized the main pattern of genetic variation in the dataset.

**FIGURE 7:**
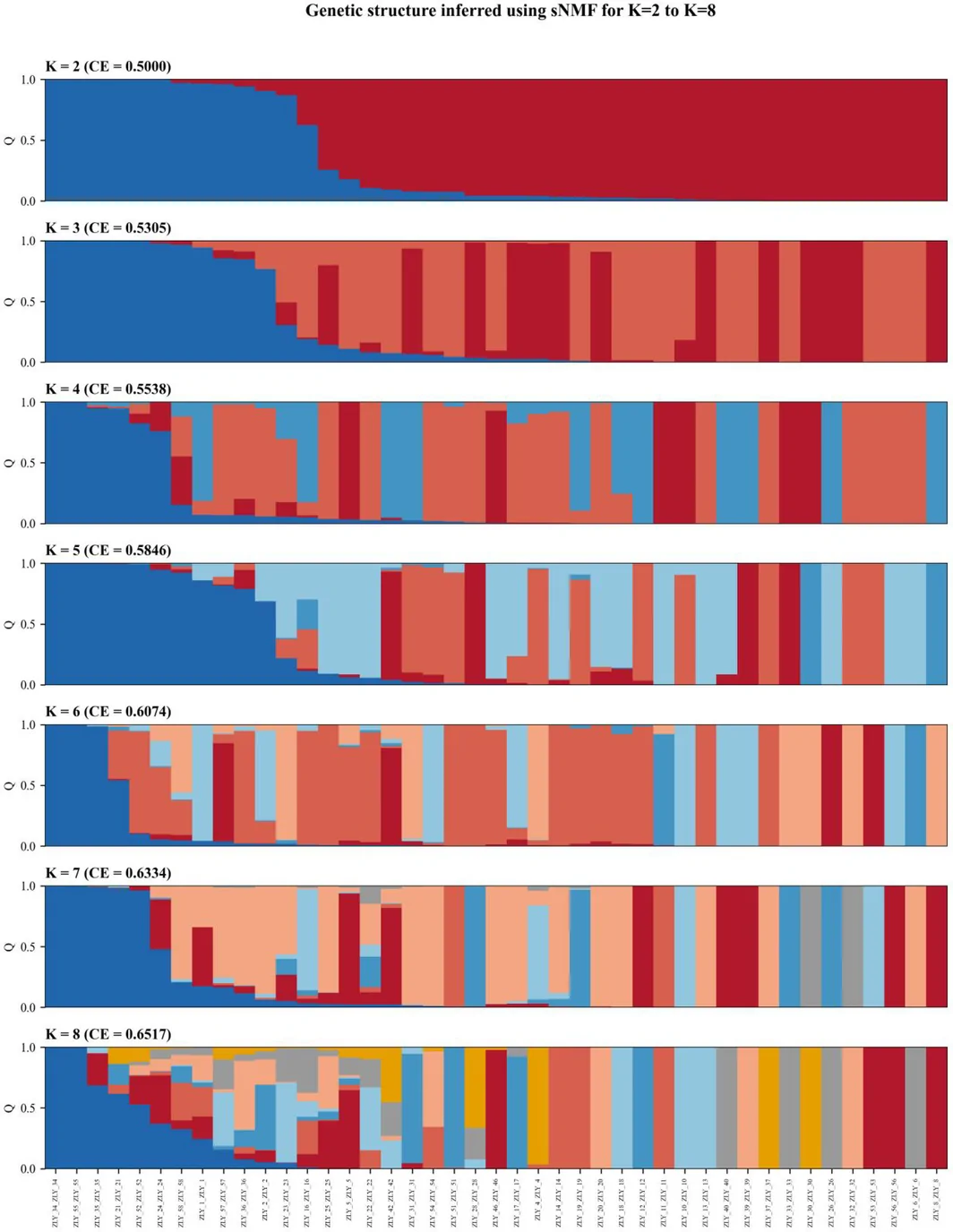
Genetic structure inferred using sNMF for *K* = 2–*K* = 8. Genetic structure inferred using sNMF for *K* = 2–*K* = 8. Each vertical bar represents one individual, and the colored segments indicate the individual coefficients for the inferred genetic components. Individuals were ordered according to their coefficient profiles within each *K*. The cross‐entropy value for each model is shown above the corresponding panel. The *K* = 2 model had the lowest cross‐entropy value (CE = 0.5000) and was therefore used as the primary representation of the major genetic structure. Higher‐*K* models were included to visualize finer‐scale variation and were not interpreted as indicating an equivalent number of discrete biological populations.

Under the *K* = 2 model, sNMF estimated two statistical ancestry components, and the individuals showed varying membership coefficients for these components. Most individuals had a predominant contribution from one component, whereas several showed appreciable contributions from both. ZLY_16 showed the most balanced assignment, with estimated membership coefficients of 0.6251 and 0.3749 for Components 1 and 2, respectively. Intermediate membership proportions were also observed in ZLY_5, ZLY_17, ZLY_31, ZLY_32, ZLY_35, ZLY_55, and ZLY_56. These individuals nevertheless showed a higher membership coefficient for one component than for the other.

The *K* = 2 model summarized two main statistical components of genetic variation, with membership coefficients varying gradually among individuals. The assignment profiles did not form two fully discrete groups. The membership coefficients describe variation in individual genetic composition, whereas the sNMF results alone do not establish recent gene flow or specific demographic processes.

Higher values of *K* subdivided the main pattern into additional components. At *K* = 3, the assignment profiles included an additional component, with predominant membership in this component observed for some individuals. At *K* = 4 and *K* = 5, further components appeared in different subsets of individuals, together with mixed membership across several components. The assignment profiles became more heterogeneous at *K* = 6 and *K* = 7, and some components were represented by relatively few individuals. The *K* = 8 model produced the most fragmented assignment pattern, with several individuals lacking a clearly predominant component.

The increasing cross‐entropy values and the greater fragmentation at higher values of *K* support *K* = 2 as the preferred model among those tested for summarizing the main pattern of genetic variation. The additional components at higher *K* represent finer differences in individual genetic composition and are not sufficient to define stable biological populations. Overall, the sNMF analysis identified two predominant statistical ancestry components and variation in their relative contributions among the sampled Tibetan antelopes.

#### 3.3.1 Kin Ship Analysis

In Figure 8 pairwise genetic relatedness was estimated for 43 Tibetan antelope individuals using 733,854 LD‐pruned autosomal SNPs distributed across chromosomes 1–29. The diagonal elements of the kinship matrix showed the highest values because they represented within‐individual comparisons and were excluded from the assessment of relationships among individuals. Most off‐diagonal elements were close to zero, as indicated by the predominantly pale background of the matrix, suggesting generally low pairwise relatedness among the sampled individuals.

**FIGURE 8:**
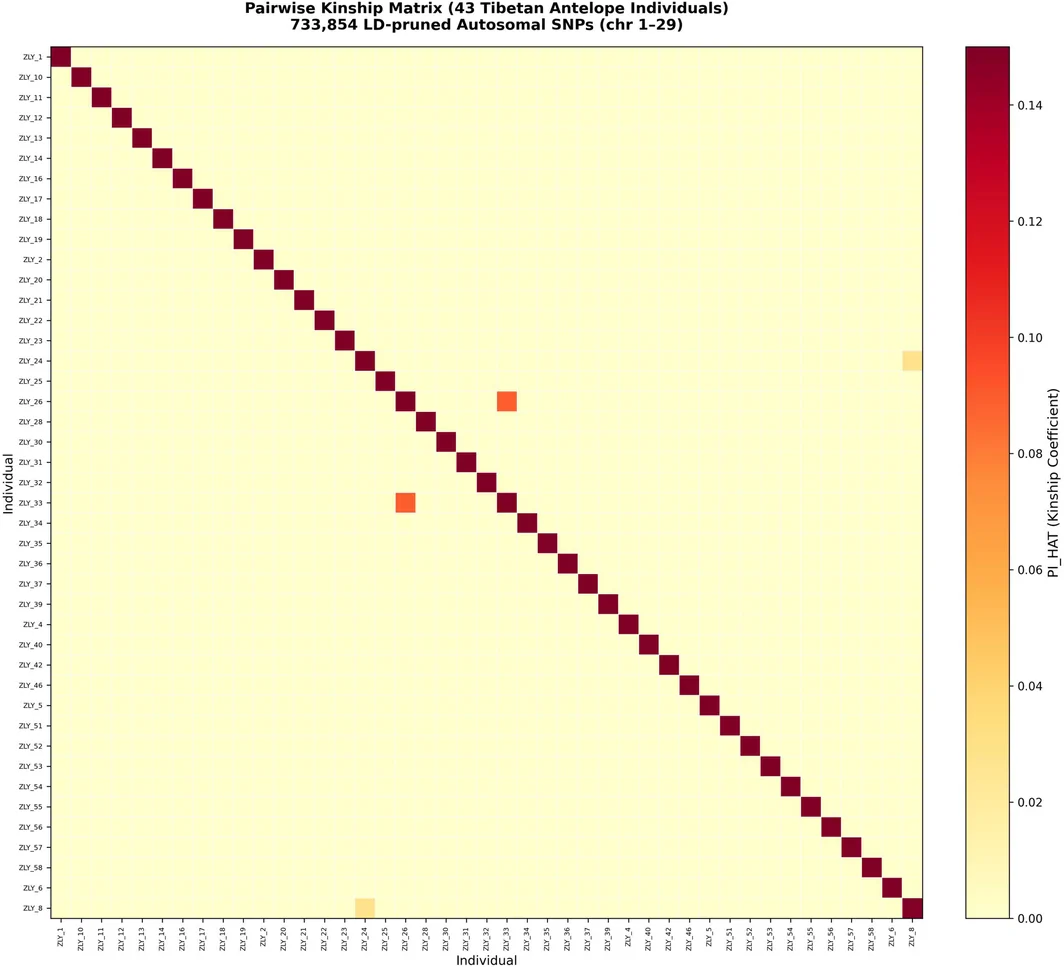
Pairwise kinship matrix for 43 Tibetan antelope individuals based on 733,013 LD‐pruned autosomal SNPs from chromosomes 1–29. The diagonal represents within‐individual comparisons. Off‐diagonal cells represent pairwise PI_HAT estimates between individuals. The color scale indicates the estimated proportion of the genome shared identical‐by‐descent.

The highest pairwise relatedness was observed between ZLY_26 and ZLY_33, with a PI_HAT value of 0.0886, followed by ZLY_24 and ZLY_8, with a PI_HAT value of 0.0274. These pairs produced the strongest off‐diagonal signals in the heatmap. Both values were below the threshold commonly used to identify second‐degree relatedness candidates, and the remaining pairwise comparisons were largely close to zero. Close relatives were therefore uncommon in the sampled dataset.

The ZLY_26‐ZLY_33 pair showed the strongest relative relatedness signal but did not fall within the range expected for first‐ or second‐degree relatives. The result indicates greater genetic similarity between these two individuals than among most other pairs, without supporting assignment to a specific pedigree category. Because the samples were obtained from naturally deceased individuals, these relatedness patterns describe the sampled dataset and may not fully represent the relatedness structure of the living population.

#### 3.3.2 Linkage Disequilibrium Analysis

LD decay was assessed using 6,991,680 QC‐filtered autosomal SNPs from chromosomes 1–29 in 43 individuals. No LD pruning was performed before the analysis. Random thinning retained 140,021, 139,969, and 140,336 SNPs in three independent replicates, corresponding to approximately 2% of the full marker set. Pairwise *r* ^2^ values were calculated for SNP pairs separated by physical distances of up to 1000 kb.

Mean *r* ^2^ declined rapidly with increasing physical distance, particularly across the shortest distance intervals, and then decreased more gradually toward 1000 kb (Figure 9). The three replicate curves closely overlapped across the analyzed distance range. In the smoothed curve, mean *r* ^2^ decreased to 0.20 at approximately 5.6 kb and to 0.10 at approximately 28.9 kb. The threshold‐based estimate of the *r* ^2^ = 0.10 distance was approximately 29.5 kb. At 0.5, 5.5, 20.5, 30.5, 50.5, and 99.5 kb, the smoothed mean *r* ^2^ values were 0.2242, 0.2003, 0.1334, 0.0959, 0.0667, and 0.0452, respectively.

**FIGURE 9:**
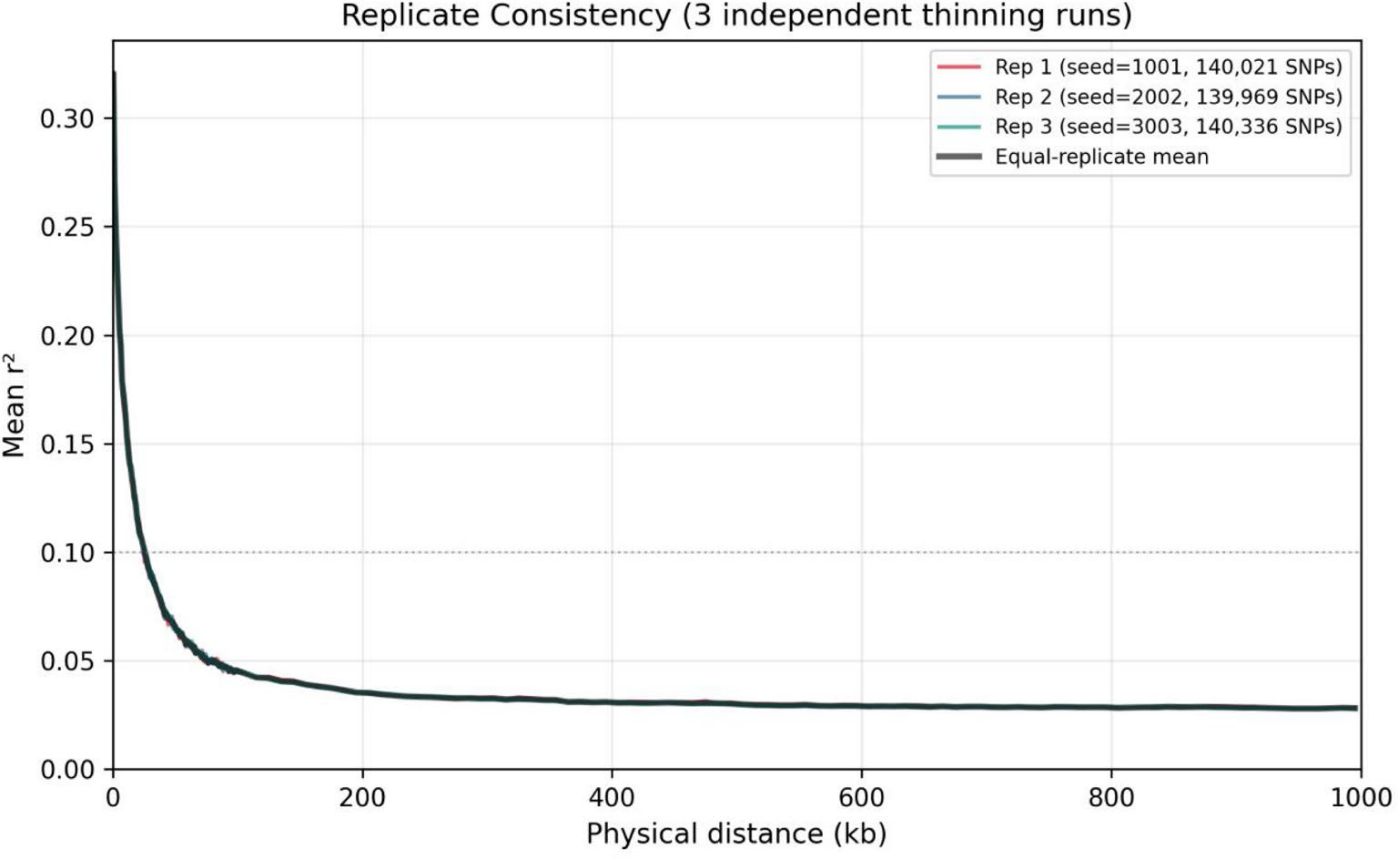
Linkage disequilibrium decay across autosomal marker distances Linkage disequilibrium decay based on 6,991,680 QC‐filtered autosomal SNPs from 43 individuals. No LD pruning was performed before the analysis. Three independent random‐thinning replicates retained approximately 2% of the marker set. Mean *r* ^2^ values are shown as a function of physical distance between SNPs, and the gray curve represents the equal‐replicate mean. The dotted horizontal line indicates *r* ^2^ = 0.10.

All 190 predefined distance bins met the minimum requirement of at least 10 valid SNP pairs per replicate, and all bins were supported by three valid replicates. The cross‐replicate standard deviation of the mean *r* ^2^ values ranged from 0.000022 to 0.004791, while the maximum absolute difference between replicate‐specific means was 0.009582. These results indicate that the overall LD decay profile was stable to random thinning of the marker set.

The observed LD decay may reflect the combined effects of recombination, effective population size, demographic history, relatedness, marker ascertainment, sampling structure, and population subdivision. The decay pattern therefore provides information relevant to LD‐based estimation of effective population size, but it does not independently demonstrate population homogeneity, current gene flow, or the absence of population structure.

#### 3.3.3 LD‐Based Effective Population Size

In Figure 10, LD‐based effective population size (*N*_e_) was estimated across a range of physical marker distances. Under the primary recombination‐rate assumption of 1.0 cM/Mb, the estimates showed a systematic decline with increasing marker distance. Shorter marker distances corresponded to older model‐based recombination timescales and yielded larger *N*_e_ estimates, whereas longer distances corresponded to more recent timescales and yielded smaller estimates.

**FIGURE 10:**
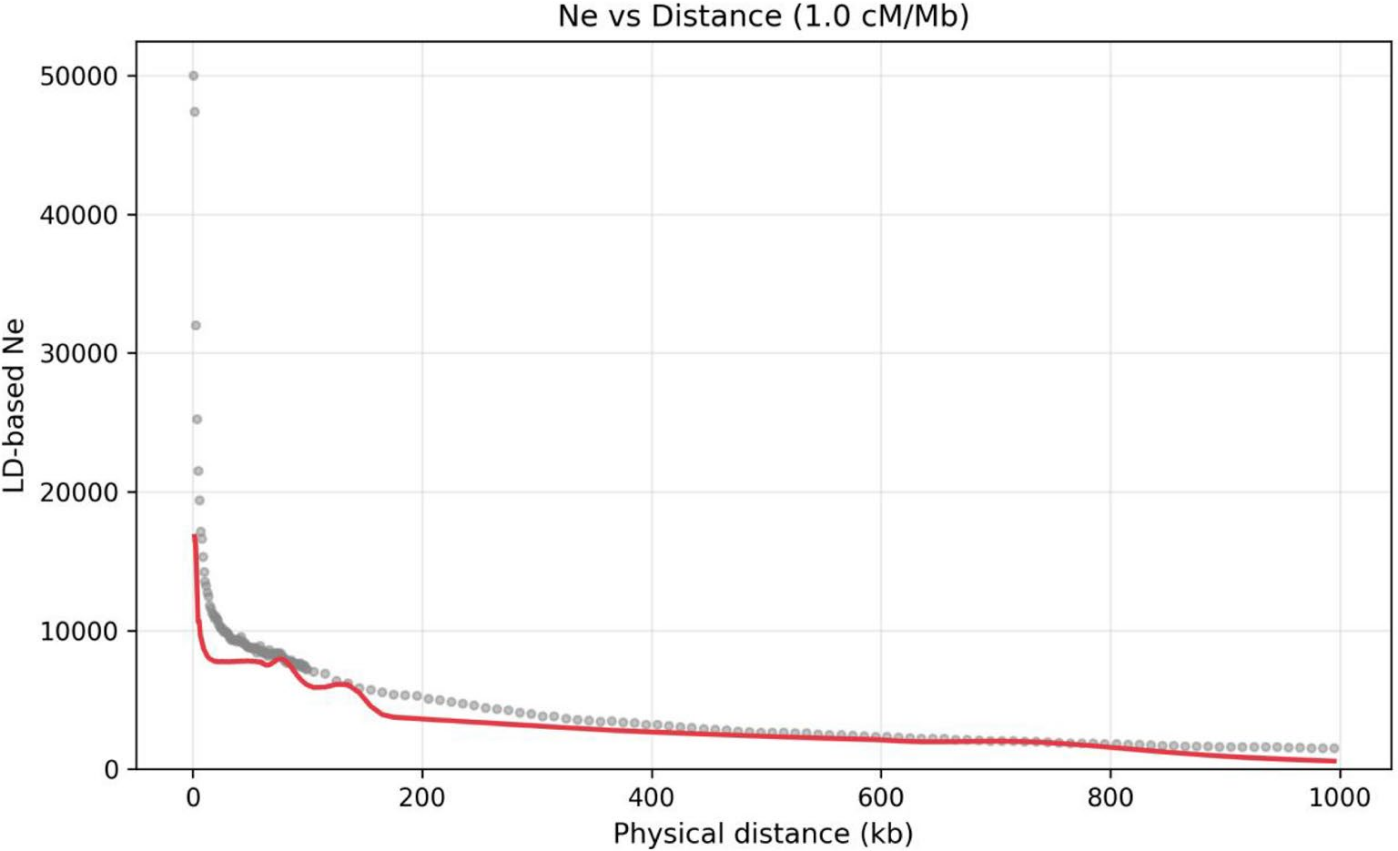
Distance‐dependent estimates of autosomal effective population size from linkage disequilibrium. Effective population size was estimated from the pooled mean. *r* ^2^ withineach physical‐distance bin using the Hill relationship and a finite‐sample correction of 1/(2*n*) = 1/86 = 0.011628, where *n* = 43.

At 1.5 kb, the estimated *N*_e_ was 47,304.15, with a replicate standard deviation of 893.53. Under the assumed recombination rate, this distance corresponded to an approximate model‐based timescale of 33,333 generations. The estimated *N*_e_ decreased to 32,171.22 at 2.5 kb, 21,508.81 at 4.5 kb, and 14,206.75 at 9.5 kb. These distances corresponded to approximately 20,000, 11,111, and 5263 generations, respectively.

At larger physical distances, the estimated *N*_e_ was 8874.56 at 49.5 kb, 7214.58 at 99.5 kb, 5129.00 ± 105.00 at 195 kb, 2674.33 at 495 kb, and 1512.14 at 995 kb. The corresponding model‐based timescales were approximately 1010, 503, 256, 101, and 50 generations, respectively.

‐The results indicate a pronounced decrease in LD‐based *N*_e_ estimates from approximately 47,304 at the oldest represented timescale to approximately 1512 at the most recent represented timescale. These values should be interpreted as historical, model‐based *N*_e_ estimates conditional on the assumed recombination rate, LD estimator, marker‐distance range, and sampled individuals. They should not be interpreted as direct estimates of census population size or as evidence of a simple, monotonic demographic trajectory without additional demographic or temporal information.

## 4 Discussion

Under the SNP dataset and ROH detection parameters used in this study, *F*_ROH_ at the 500‐kb minimum ROH threshold ranged from 0.0022 to 0.0351, with a mean of 0.0114 and a median of 0.0081. The Serling Tso Tibetan antelope samples therefore showed a relatively low proportion of the genome covered by ROHs, although considerable variation occurred among individuals. Reducing the minimum ROH threshold from 1000 to 100 kb increased *F*_ROH_, ROH number, and total ROH length. Long ROHs contributed relatively little to total ROH coverage. Under the ROH detection settings used here, short and medium‐length ROHs accounted for most of the detected ROH coverage, whereas long ROHs contributed relatively little.

ROH length reflects the age of common ancestry and the history of recombination. Segments inherited from recent common ancestors experience fewer recombination events and tend to remain long. Older shared segments are progressively shortened over successive generations. The limited contribution of long ROHs provides little evidence of extensive recent autozygosity among the sampled individuals under the detection settings used here. Short and medium‐length ROHs may reflect historical common ancestry, long‐term genetic drift, or past changes in effective population size. ROH length categories and minimum detection thresholds can substantially affect ROH number, total ROH length, and *F*_ROH_ estimates, as shown in studies of Moroccan indigenous sheep and Iranian fat‐tailed sheep (Kusza et al. 2024; Abdoli et al. 2023). The present results follow the general pattern reported in Moroccan indigenous sheep and Iranian fat‐tailed sheep. In these studies, short‐, medium‐, and long‐length ROHs were used to examine common ancestry and inbreeding across different time scales. As in the present analysis, lowering the minimum detectable ROH length can increase the number and total length of detected ROHs, thereby changing *F*_ROH_ estimates (Kusza et al. 2024; Abdoli et al. 2023). Livestock populations are shaped by artificial selection, closed breeding systems, and managed mating. These studies therefore provide methodological references for interpreting ROH patterns in Tibetan antelopes.

The Tibetan antelope samples had a lower contribution from long ROHs than the Scandinavian wolf population and a greater representation of short and medium‐length ROHs. In Scandinavian wolves, long ROHs and extensive genome‐wide IBD sharing were associated with recent common ancestry, prolonged isolation, and inbreeding (Kardos et al. 2024; Smeds et al. 2024).

The difference between the two species suggests distinct demographic histories and different strengths of recent inbreeding signals. Direct quantitative comparison remains inappropriate because the studies used different species, sampling designs, SNP densities, and ROH detection thresholds.

LD declined with increasing physical distance between markers, and the same pattern appeared across thinning replicates. The samples therefore showed a consistent distance‐dependent LD decay pattern. Recombination contributed to the reduction of LD between distant markers, while population size, genetic drift, population structure, and admixture may also have influenced LD levels. The LD measurements were also used for the LD‐based *N*_e_ analysis reported in this study; the distance‐dependent decay pattern alone does not establish the magnitude or direction of *N*_e_ change. LD decay alone does not provide the direction of *N*_e_ change.

ROH and LD‐based *N*_e_ describe different aspects of population history. ROH analysis focuses on continuous homozygous segments within individuals and their possible historical origins. LD‐based *N*_e_ estimates are sensitive to the sampled individuals and analytical settings, including population structure and marker filtering. ROH and LD‐based *N*_e_ therefore provide complementary summaries of the analyzed sample set, without independently resolving a specific demographic history. Future analyses should report *N*_e_ estimates and confidence intervals for different marker‐distance intervals and evaluate the effects of population structure, allele‐frequency thresholds, and sample size.

PCA and sNMF revealed genetic structure among the samples, while individual ancestry profiles showed substantial overlap. Kinship analysis indicated that pairwise relatedness was close to zero for most individuals, and no first‐ or second‐degree relationships were detected. These results show variation in individual genetic composition and overlapping sNMF membership coefficients within the analyzed sample set. No first‐ or second‐degree relatives were detected among the sampled individuals.

The *K* = 2 ancestry pattern provides a simplified description of the genetic structure in the present sample. The overlapping ancestry profiles show that the two statistical components estimated at *K* = 2 do not define two fully discrete groups in this sample. Geographic distance, individual dispersal, and habitat configuration may also have contributed to this pattern. PCA and sNMF results alone do not determine the timing or direction of gene flow.

This interpretation is consistent with genomic research on Asian wolves, which identified geographic differentiation alongside complex historical connections (Hendricks et al. 2025; Salado et al. 2024). Studies of gray wolf genomic history have likewise shown that population structure can reflect historical admixture and ancestral exchange (Stewart et al. 2025). These studies provide comparative support for interpreting the genetic structure observed in Tibetan antelopes, although differences in migration behavior, social organization, mating system, and demographic history should be considered.

Heterozygosity mainly reflects allele‐frequency composition, whereas PIC describes the polymorphism and information content of individual markers. ROH and *F*_ROH_ characterize continuous homozygous segments and individual‐level genomic inbreeding. These indices therefore describe different components of genetic variation. The PIC values describe the allele‐frequency distribution and informativeness of the analyzed SNP markers; they do not, on their own, establish the genetic health of the sampled animals. PIC can be influenced by rare‐allele frequency, marker selection, sequencing platform, and sample size. Studies of ROH commonly combine heterozygosity, ROH, relatedness, and population structure to assess genetic status (Rees et al. 2026). Previous genomic studies provide important reference resources for the Tibetan antelope. Ge et al. (2013) reported a draft genome sequence of the species, whereas Xu et al. (2024) produced a high‐quality chromosome‐level reference genome assembly of Tibetan antelope ( *Pantholops hodgsonii* ). These studies provide genomic resources rather than directly comparable population‐level estimates of PIC, heterozygosity, ROH, or relatedness. The present study complements these resources by reporting SNP‐based estimates of marker informativeness, heterozygosity, autozygosity, LD, relatedness, and individual genetic composition for Tibetan antelopes sampled from the Selincuo region. Because the study objectives, sampling designs, and analytical methods differ, the results should not be compared quantitatively. Instead, the present findings provide a regional population‐genomic baseline. Overall, the Serling Tso Tibetan antelope samples showed no strong signal of severe recent inbreeding under the analytical framework used here. Several individuals had relatively high inbreeding‐related estimates and should receive continued attention in future monitoring. The predominance of short and medium‐length ROHs indicates that historical demographic processes contributed substantially to genomic homozygosity. PCA and sNMF showed variation in genetic composition among the sampled individuals, with substantial overlap in their ancestry profiles. Kinship analysis indicated detectable relatedness in some pairs, but most individuals were distantly related, and no first‐ or second‐degree relationships were detected.

This study provides a preliminary genomic baseline for Tibetan antelopes sampled from the Selincuo region. The baseline can support future monitoring of *F*_ROH_, ROH length distributions, LD‐based *N*_e_, relatedness, and population structure. The current spatial and temporal sampling coverage limits the reconstruction of historical bottlenecks, long‐term drift, recent gene flow, and local mating structure. Future studies should expand sampling across geographic regions and time periods and integrate whole‐genome sequencing, IBD analysis, spatial genetic analysis, genome‐wide FST, isolation‐by‐distance tests, gene‐flow reconstruction, and demographic modeling. Linking *F*_ROH_ with individual fitness, disease susceptibility, and reproductive performance will help determine whether genomic homozygosity affects the adaptive potential and conservation management of Tibetan antelopes.

## 5 Conclusion

Short ROH segments predominated, whereas long segments contributed relatively little to total ROH coverage. This pattern may reflect older shared ancestry, historical demographic changes, and background genetic drift. Individual differences in ROH profiles indicate variation in autozygosity within the sampled population, with some individuals potentially carrying a greater burden of homozygous segments.

The population retained considerable genome‐wide SNP variation, although allele frequencies were uneven and low‐ to intermediate‐frequency variants were relatively common. The modest heterozygote deficit may reflect several interacting processes, including population structure, historical relatedness, genetic drift, and demographic change. Inbreeding could also contribute to this pattern, although the present data do not allow its relative importance to be determined. PCA and sNMF showed broadly consistent patterns of genetic differentiation among individuals. The inferred ancestry components may represent genetic heterogeneity within the sampled population, but their biological significance remains uncertain. The current sampling design cannot establish whether these components correspond to persistent geographic groups or formal management units.

The genomic profile of the Selincuo Tibetan antelope population includes retained genetic variation, moderate genetic structuring, and individual differences in homozygosity. The available results provide limited evidence for severe genetic erosion across the sampled population. Several limitations should be considered. The study included one population and a relatively small number of individuals, and it lacked direct comparisons with geographically matched populations. The sampling design also limits the assessment of spatial genetic variation and may influence estimates of population structure, relatedness, and LD. In addition, each genomic indicator reflects a different aspect of genetic variation. Heterozygosity describes allele and genotype variation, *F*_ROH_ reflects homozygous segments and their length distribution, *F*_PLINK_ is influenced by allele frequencies and marker composition, PIC mainly measures marker informativeness, and LD decay reflects linkage patterns shaped by recombination and demographic history. These measures therefore require joint interpretation.

The present analyses do not directly estimate migration rates, gene flow, or genetic connectivity among protected areas. The observed PCA and sNMF patterns may be compatible with partial connectivity, although this interpretation cannot be confirmed with the current dataset. Future research should include geographically separated Tibetan antelope populations and evaluate genetic connectivity among protected areas using genome‐wide F_ST_, pairwise relatedness, IBD sharing, isolation‐by‐distance analyses, admixture patterns, and direct estimates of migration or gene flow. Such comparisons could clarify whether the observed genetic variation represents local differentiation within a connected population or more persistent geographic structuring.

Further studies should also examine nucleotide diversity, ROH length classes, effective population size, and the geographic distribution of rare alleles. These analyses would help determine how historical drift, demographic change, population structure, and recent inbreeding have contributed to the genomic characteristics observed in Tibetan antelopes sampled from the Selincuo region.

## Author Contributions

**Siwei Gao:** data curation (equal), investigation (equal), methodology (equal), writing – original draft (equal). **Yibo Zhang:** data curation (equal), investigation (equal). **Zhonghao Yang:** data curation (equal), investigation (equal). **Yanling Zhao:** project administration (equal), supervision (equal). **Xin Song:** data curation (equal), writing – review and editing (equal). **Wentao Kong:** data curation (equal), writing – review and editing (equal). **Zili Ren:** funding acquisition (lead), project administration (lead), writing – review and editing (lead).

## Funding

This work was supported by the State Key Laboratory of Microbial Technology Open Projects Fund (project nos. M2024‐03 and M2024‐04); Postgraduate Education Innovation Program of Xizang Agricultural and Animal Husbandry University (DKYC2026010); Science and Technology Program of Xizang Autonomous Region (XZ202402ZY0025); “Qomolangma Talents” Science and Technology Innovation Team of Xizang Autonomous Region (2025.08–2030.08); Regional Fund Project of National Natural Science Foundation of China (32660927); University‐Local Cooperation Project of Xizang Agricultural and Animal Husbandry University supported by Nyingchi Science and Technology Bureau (XDCG202601).

## Conflicts of Interest

The authors declare no conflicts of interest.

## Supporting information


**Data S1:** ece374598‐sup‐0001‐Supinfo.zip.

## Data Availability

All relevant data have been deposited in the China National Center for Bioinformation under accession number CRA045501. All raw data will be made publicly available upon publication of the manuscript.

## References

[ece374598-bib-0001] Abdoli, R. , S. Z. Mirhoseini , N. Ghavi Hossein‐Zadeh , et al. 2023. “Runs of Homozygosity and Cross‐Generational Inbreeding of Iranian Fat‐Tailed Sheep.” Heredity 130, no. 6: 358–367. 10.1038/s41437-023-00611-y PMC10238534 37016136

[ece374598-bib-0002] Anderson, C. A. , F. H. Pettersson , G. M. Clarke , L. R. Cardon , A. P. Morris , and K. T. Zondervan . 2010. “Data Quality Control in Genetic Case–Control Association Studies.” Nature Protocols 5, no. 9: 1564–1573. 10.1038/nprot.2010.116. PMC3025522 21085122

[ece374598-bib-0003] Andersson, R. , C. Gebhard , I. Miguel‐Escalada , et al. 2010. “FastQC: A Quality Control Tool for High Throughput Sequence Data.” Version 0.11, 2.

[ece374598-bib-0004] Botstein, D. , R. L. White , M. Skolnick , and R. W. Davis . 1980. “Construction of a Genetic Linkage Map in Man Using Restriction Fragment Length Polymorphisms.” American Journal of Human Genetics 32, no. 3: 314–331. PMC1686077 6247908

[ece374598-bib-0005] Ceballos, F. C. , P. K. Joshi , D. W. Clark , M. Ramsay , and J. F. Wilson . 2018. “Runs of Homozygosity: Windows Into Population History and Trait Architecture.” Nature Reviews Genetics 19, no. 4: 220–234. 10.1038/nrg.2017.109. 29335644

[ece374598-bib-0006] Chen, S. , Y. Zhou , Y. Chen , and J. Gu . 2018. “Fastp: An Ultra‐Fast All‐in‐One FASTQ Preprocessor.” Bioinformatics 34, no. 17: i884–i890. 10.1093/bioinformatics/bty560. PMC6129281 30423086

[ece374598-bib-0007] Ge, R. L. , Q. Cai , Y. Y. Shen , et al. 2013. “Draft Genome Sequence of the Tibetan Antelope.” Nature Communications 4, no. 1: 1858. 10.1038/ncomms2860 PMC3674232 23673643

[ece374598-bib-0008] Keller, M. C. , P. M. Visscher , and M. E. Goddard . 2011. “Quantification of Inbreeding due to Distant Ancestors and Its Detection Using Dense Single Nucleotide Polymorphism Data.” Genetics 189, no. 1: 237–249. 10.1534/genetics.111.130922. PMC3176119 21705750

[ece374598-bib-0009] Kusza, S. , B. Badaoui , and G. Wanjala . 2024. “Insights Into the Genomic Homogeneity of Moroccan Indigenous Sheep Breeds Though the Lens of Runs of Homozygosity.” Scientific Reports 14, no. 1: 16515. 10.1038/s41598-024-67558-w. PMC11255268 39019985

[ece374598-bib-0010] McQuillan, R. , A. L. Leutenegger , R. Abdel‐Rahman , et al. 2008. “Runs of Homozygosity in European Populations.” American Journal of Human Genetics 83, no. 3: 359–372. 10.1016/j.ajhg.2008.08.007 PMC2556426 18760389

[ece374598-bib-0011] Nei, M. 1973. “Analysis of Gene Diversity in Subdivided Populations.” Proceedings of the National Academy of Sciences 70, no. 12: 3321–3323. 10.1073/pnas.70.12.3321. PMC427228 4519626

[ece374598-bib-0012] Onzima, R. B. , H. P. Doekes , L. F. Brito , et al. 2018. “Genome‐Wide Characterization of Runs of Homozygosity and Estimation of Genomic Inbreeding in Ugandan Goat Breeds.” *Proceedings of the World Congress on Genetics Applied to Livestock Production*.

[ece374598-bib-0013] Patterson, N. , A. L. Price , and D. Reich . 2006. “Population Structure and Eigenanalysis.” PLoS Genetics 2, no. 12: e190. 10.1371/journal.pgen.0020190. PMC1713260 17194218

[ece374598-bib-0014] Price, A. L. , N. J. Patterson , R. M. Plenge , M. E. Weinblatt , N. A. Shadick , and D. Reich . 2006. “Principal Components Analysis Corrects for Stratification in Genome‐Wide Association Studies.” Nature Genetics 38, no. 8: 904–909. 10.1038/ng1847. 16862161

[ece374598-bib-0015] Purcell, S. , B. Neale , K. Todd‐Brown , et al. 2007. “PLINK: A Tool Set for Whole‐Genome Association and Population‐Based Linkage Analyses.” American Journal of Human Genetics 81, no. 3: 559–575. 10.1086/519795 PMC1950838 17701901

[ece374598-bib-0016] Purfield, D. C. , D. P. Berry , S. McParland , and D. G. Bradley . 2012. “Runs of Homozygosity and Population History in Cattle.” BMC Genetics 13, no. 1: 70. 10.1186/1471-2156-13-70. PMC3502433 22888858

[ece374598-bib-0017] Rees, J. , M. Sherman , D. Teofilov , et al. 2026. “Joint Dog and Wolf Genealogies Reveal the Evolution of the Canine Genome.” *bioRxiv*: The Preprint Server for Biology, 2026‐06.

[ece374598-bib-0018] Rosenberg, N. A. , T. J. Pemberton , J. Z. Li , and J. W. Belmont . 2013. “Runs of Homozygosity and Parental Relatedness.” Genetics in Medicine 15, no. 9: 753–754. 10.1038/gim.2013.108. 24008258

[ece374598-bib-0019] Salado, I. , M. Preick , N. Lupiáñez‐Corpas , et al. 2024. “Large Variance in Inbreeding Within the Iberian Wolf Population.” Journal of Heredity 115, no. 4: 349–359. 10.1093/jhered/esad071. PMC11235127 37955431

[ece374598-bib-0020] Schaller, G. B. 2000. Wildlife of the Tibetan Steppe. University of Chicago Press.

[ece374598-bib-0021] Smeds, L. , L. S. Huson , and H. Ellegren . 2024. “Structural Genomic Variation in the Inbred Scandinavian Wolf Population Contributes to the Realized Genetic Load but Is Positively Affected by Immigration.” Evolutionary Applications 17, no. 2: e13652. 10.1111/eva.13652 PMC10848878 38333557

[ece374598-bib-0022] Smith, A. T. , D. Lunde , R. S. Hoffmann , et al. 2010. A Guide to the Mammals of China. Princeton University Press.

[ece374598-bib-0023] Stewart, M. , C. Peters , M. J. Ziegler , et al. 2025. “The State of the Late Quaternary Megafauna Extinction Debate: A Systematic Review and Analysis.” Frontiers in Mammal Science 4: 1678231. 10.3389/fmamm.2025.1678231.

[ece374598-bib-0024] VanRaden, P. M. 2008. “Efficient Methods to Compute Genomic Predictions.” Journal of Dairy Science 91, no. 1: 414–4423. 10.3168/jds.2007-0980 18946147

[ece374598-bib-0025] Waples, R. S. , and C. H. I. Do . 2010. “Linkage Disequilibrium Estimates of Contemporary Ne Using Highly Variable Genetic Markers: A Largely Untapped Resource for Applied Conservation and Evolution.” Evolutionary Applications 3, no. 3: 244–262. 10.1111/j.1752-4571.2009.00104.x. PMC3352464 25567922

[ece374598-bib-0026] Waples, R. S. , and P. R. England . 2011. “Estimating Contemporary Effective Population Size on the Basis of Linkage Disequilibrium in the Face of Migration.” Genetics 189, no. 2: 633–644. 10.1534/genetics.111.132233. PMC3189803 21840864

[ece374598-bib-0027] Wigginton, J. E. , D. J. Cutler , and G. R. Abecasis . 2005. “A Note on Exact Tests of Hardy–Weinberg Equilibrium.” American Journal of Human Genetics 76, no. 5: 887–893. 10.1086/429864. PMC1199378 15789306

[ece374598-bib-0028] Xu, B. , J. Chen , P. Song , et al. 2024. “A High‐Quality Chromosome‐Level Reference Genome Assembly of Tibetan Antelope ( *Pantholops hodgsonii* ).” Scientific Data 11, no. 1: 1215. 10.1038/s41597-024-04089-z. PMC11557879 39532915

